# Comparative analysis of the surface exposed proteome of two canine osteosarcoma cell lines and normal canine osteoblasts

**DOI:** 10.1186/1746-6148-9-116

**Published:** 2013-06-13

**Authors:** Milan Milovancev, Ian Hilgart-Martiszus, Michael J McNamara, Cheri P Goodall, Bernard Seguin, Shay Bracha, Samanthi I Wickramasekara

**Affiliations:** 1 Department of Clinical Sciences, College of Veterinary Medicine, Oregon State University, Corvallis, OR 97331, USA; 2 EACRI, Providence Portland Medical Center, Portland, OR 97213, USA; 3 Department of Chemistry, Oregon State University, Corvallis, OR 97331, USA; 4 Present address: The Animal Cancer Center, College of Veterinary Medicine and Biomedical Sciences, Colorado State University, Fort Collins, CO 80523, USA

**Keywords:** Dog, Proteomics, Osteosarcoma, Mass spectrometry, Biotinylation

## Abstract

**Background:**

Osteosarcoma (OSA) is the most common primary bone tumor of dogs and carries a poor prognosis despite aggressive treatment. An improved understanding of the biology of OSA is critically needed to allow for development of novel diagnostic, prognostic, and therapeutic tools. The surface-exposed proteome (SEP) of a cancerous cell includes a multifarious array of proteins critical to cellular processes such as proliferation, migration, adhesion, and inter-cellular communication. The specific aim of this study was to define a SEP profile of two validated canine OSA cell lines and a normal canine osteoblast cell line utilizing a biotinylation/streptavidin system to selectively label, purify, and identify surface-exposed proteins by mass spectrometry (MS) analysis. Additionally, we sought to validate a subset of our MS-based observations via quantitative real-time PCR, Western blot and semi-quantitative immunocytochemistry. Our hypothesis was that MS would detect differences in the SEP composition between the OSA and the normal osteoblast cells.

**Results:**

Shotgun MS identified 133 putative surface proteins when output from all samples were combined, with good consistency between biological replicates. Eleven of the MS-detected proteins underwent analysis of gene expression by PCR, all of which were actively transcribed, but varied in expression level. Western blot of whole cell lysates from all three cell lines was effective for Thrombospondin-1, CYR61 and CD44, and indicated that all three proteins were present in each cell line. Semi-quantitative immunofluorescence indicated that CD44 was expressed at much higher levels on the surface of the OSA than the normal osteoblast cell lines.

**Conclusions:**

The results of the present study identified numerous differences, and similarities, in the SEP of canine OSA cell lines and normal canine osteoblasts. The PCR, Western blot, and immunocytochemistry results, for the subset of proteins evaluated, were generally supportive of the mass spectrometry data. These methods may be applied to other cell lines, or other biological materials, to highlight unique and previously unrecognized differences between samples. While this study yielded data that may prove useful for OSA researchers and clinicians, further refinements of the described techniques are expected to yield greater accuracy and produce a more thorough SEP analysis.

## Background

Osteosarcoma (OSA) is the most common primary bone tumor of dogs with >8000 cases diagnosed annually in the United States of America [1]. Despite aggressive treatment incorporating surgery, radiation therapy, and/or chemotherapy, survival times remain poor. Over 90% of canine patients undergoing standard-of-care treatment, without clinically detectable metastasis at time of diagnosis, will ultimately succumb to metastatic disease [1,2]. Overall reported median survival times are 235-366 days, and 1- and 2-year survival rates range from 33-65% and 16-28%, respectively [1-4]. The development of effective tools to diagnose and treat OSA is essential for mitigating the negative impact of this disease on both canine patients and their owners.

The extracellular surface of a cancerous cell includes a multifarious array of proteins that are critical to processes such as cell proliferation, migration, adhesion, and inter-cellular communication. Collectively, this group of proteins can be referred to as the surface-exposed proteome (SEP). Abnormalities in the SEP are central to the biologic behavior of cancer [5]. Recognized abnormalities in cancerous cells include changes in the expression level of particular proteins, the presence of unusual protein isoforms, and alterations in post-translational modification patterns (e.g. phosphorylation or glycosylation) [5,6].

Despite OSA’s considerable clinical impact, a comparative analysis of the SEP between canine osteosarcoma and normal osteoblast cells has not been described in the veterinary literature. Isolated reports of specific abnormalities in canine OSA are limited to focused evaluations of specific proteins, often prompted by discoveries in other neoplasms or species. Examples of such proteins include: survivin [7], cathepsin K [8], Met [9-11], EGFR [9], Ron [9], p53 [12,13], HER-2 [14], HGF [10,11], IGF-1 and IGF-1R [15], MMP-2 and MMP-9 [16,17], and ezrin [18]. A more global analysis of the SEP differences between OSA and normal osteoblast cells holds the potential of identifying unique and previously unrecognized features of OSA that can be exploited in treatment, diagnostic, and/or prognostic capacities [5,6,19-23].

While there are many different methods available for enriching plasma membrane proteins, labeling surface proteins with biotin is a popular approach because of the availability of membrane-impermeable biotinylation reagents and the strong association between biotin and streptavidin, which is used for affinity purification of biotin-labeled molecules [24]. These two attributes make it feasible to selectively label surface proteins, and then use denaturing conditions to extract total protein from labeled cells and affinity purify the biotinylated fraction. The purified surface proteins can then be enzymatically digested with trypsin and the resulting peptides identified by mass spectrometry (MS). MS-based proteomics has been applied to numerous cancer biomarker discovery projects and enables protein-level investigations reflecting many of the biological processes relevant to cancer growth and invasion [20,21,25,26].

The specific aim of our study was to define a profile of proteins that compose the SEP of two validated canine OSA cell lines and a normal canine osteoblast cell line utilizing the aforementioned biotinylation/streptavidin system to selectively label, purify, and identify surface-exposed proteins by LC-MS/MS analysis. Additionally, we sought to validate a subset of our MS-based observations via quantitative real-time PCR (qRT-PCR) and antibody-based techniques. Our hypothesis was that MS would detect differences in the SEP composition between the OSA and the cultured normal osteoblast cells.

## Results

### Identification of surface-exposed proteins

**Figure 1:**
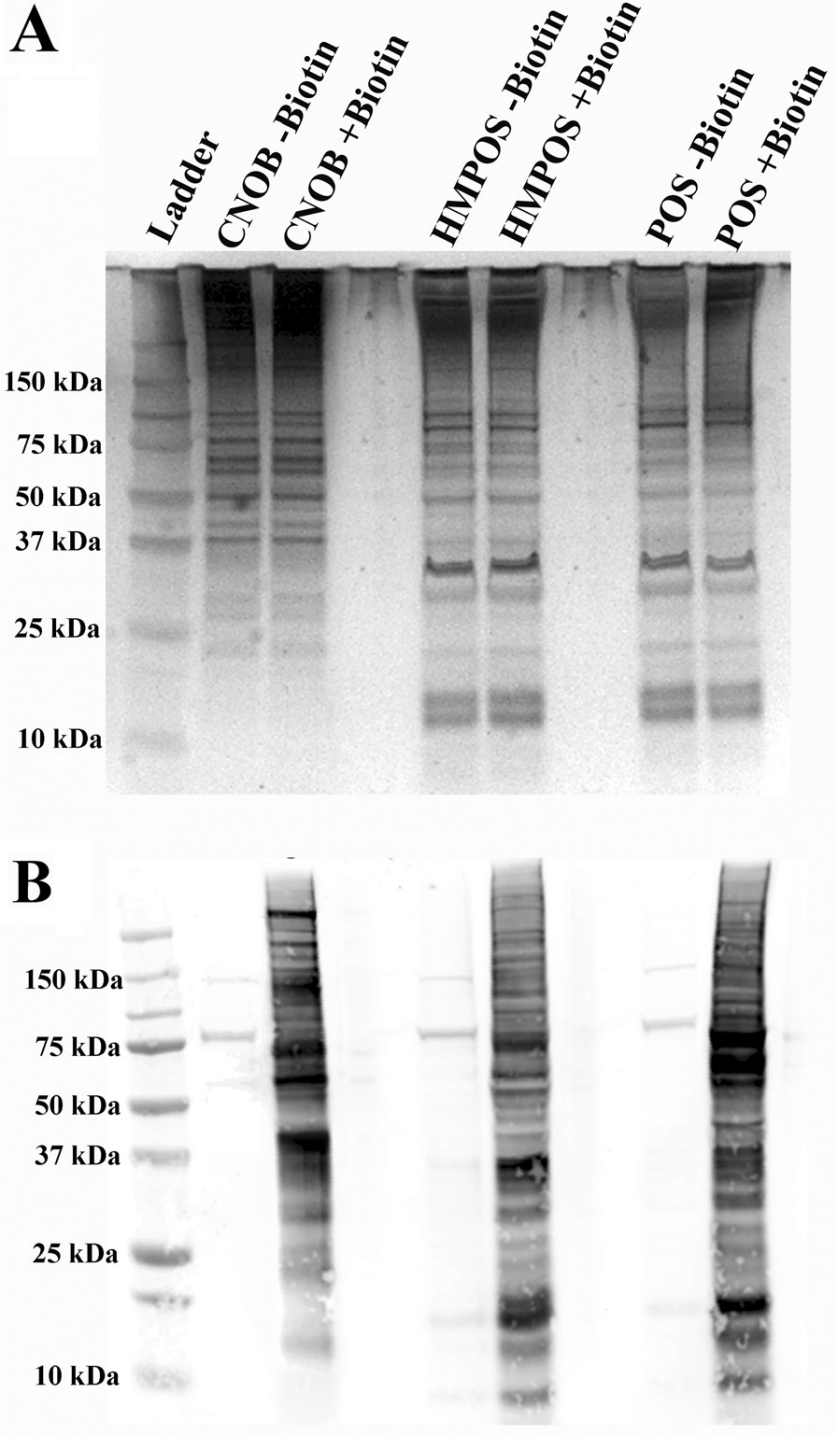
**Biotinylation of cell lines.** (**A**) Silver stain of total protein from biotin treated and non-biotin treated cultured normal canine osteoblasts (CnOb) and two validated canine osteosarcoma cell lines (POS and HMPOS). (**B**) Anti-biotin Western blot of all biotinylated proteins from biotin treated and non-biotin treated CnOb, POS, and HMPOS cell line samples.

The biotin-labeled surface proteins from each cell line were extracted and visualized by SDS-PAGE and Western blot (Figure 1). The gross comparison of biotinylation patterns indicated that the complement of surface-exposed proteins from the POS and HMPOS cell lines appeared similar, while the surface proteome of the CnOb cell line appeared to be markedly different. Following the affinity purification and proteolysis of the biotinylated proteins, analysis of the peptides by shotgun MS identified a total of 133 putative surface proteins when outputs from all samples and replicates were combined (Additional file 1: Table S1). Protein identifications were largely consistent between biological replicates (Additional file 2: Table S2). Based on their potential relevance to cancer biology, a subset of the identified proteins was selected for inclusion in Table 1, with their relative spectral counts expressed as a percentage of total spectra. Several proteins that are known to be associated with the cell surface and/or interact with the extracellular matrix were detected in all three cell lines, such as Fibronectin, Vitronectin, CYR61 and Annexin A2 [27-30]. Several additional surface proteins were found to be detected in abundance in one cell line, but poorly detected or absent in the others. For example, peptides originating from Thrombospondin-1 were observed in abundance from CnOb samples, but were not observed in any of the replicates from the POS or HMPOS cell lines. Similarly, both Chondroitin Sulfate Proteoglycan 4 (CSPG4) and CD109 were detected from POS samples, but were not detected in the other samples.

**Table 1:** Subset of mass spectrometry-identified surface proteins of cell lines

**Protein name**	**Gene name**	**NCBI Ref Sequence**	**CnOb**	**POS**	**HMPOS**
Fibronectin	FN1	XP_536059	0.55	0.16	0.14
Annexin 2	ANXA2	NP_001002961	0.15	0.33	0.43
Protein CYR61	CYR61	XP_537091	0.1	0.01	0.48
Vitronectin	VTN	XP_854040	0.1	0.11	0.13
Serpin Peptidase Inhibitor	SERPINH1	NP_001159360	0.17	0.37	0.24
Thrombospondin-1	THBS1	XP_544610	1.1	0	0
Chondroitin sulfate proteoglycan 4	CSPG4	XP_544783	0	2.0	0
CD109 antigen isoform 3	CD109	XP_532205	0	0.21	0
Neuropilin-1 isoform 2	NRP1	XP_535142	0	0.12	0
Glypican-4	GPC4	XP_549265	0	0.11	0
4 F2 cell-surface antigen heavy chain	SLC3A2	XP_540898	0	0.11	0
Cysteine-rich secretory protein LCCL domain-containing 2	CRISPLD2	XP_546797	0	0	0.06
Integrin Beta-1	ITGB1	XP_535143	0.07	0.08	0
Plexin B2	PLXNB2	XP_531689	0	0.77	0.11
CD44 antigen precursor	CD44	NP_001183951	0	0.15	0.07
Ephrin type-A receptor 2 isoform 1	EPHA2	XP_544546	0	0.35	0.06
Inactive tyrosine-protein kinase 7 isoform A	PTK7	XP_538929	0	0.42	0.02
C-type mannose receptor 2	MRC2	XP_003435244	0	0.22	0.01
Delta-sarcoglycan	SGCD	XP_854897	0	0.16	0.01
Notch homolog protein 2 isoform 1	NOTCH2	XP_540266	0	0.08	0.02

A subset of the surface-exposed proteins identified by mass spectrometry in cultured normal canine osteoblasts (CnOb) and two validated canine osteosarcoma cell lines (POS and HMPOS), with their relative spectral counts expressed as a percentage of total spectra.

### qRT-PCR

**Figure 2:**
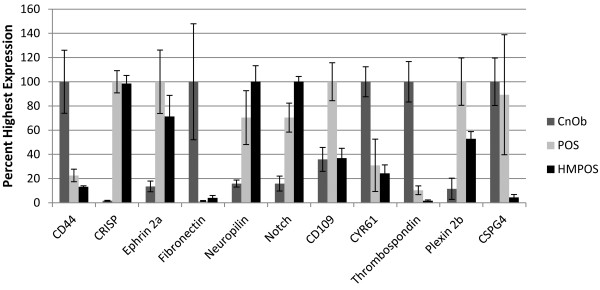
**Quantitative real-time PCR of cell lines.** Quantitative real-time PCR indicating relative expression of selected genes corresponding to surface proteins detected by mass spectrometry of cultured normal canine osteoblasts (CnOb) and two validated canine osteosarcoma cell lines (POS and HMPOS).

Eleven of the proteins that were detected by MS were selected for secondary analysis of gene expression by qRT-PCR, the results of which are summarized in Figure 2. These eleven proteins were selected on the basis of their relevance to cancer biology and the availability of antibodies that were potentially compatible with canine targets. Active transcription of all of the selected genes was detected by qRT-PCR, indicating that all of these genes are indeed expressed in the cell lines used in this study. Expression was normalized to GAPDH and the biological replicates were generally in close agreement between samples, with few minor variations (Additional file 3: Table S3).

### Western blot

**Figure 3:**
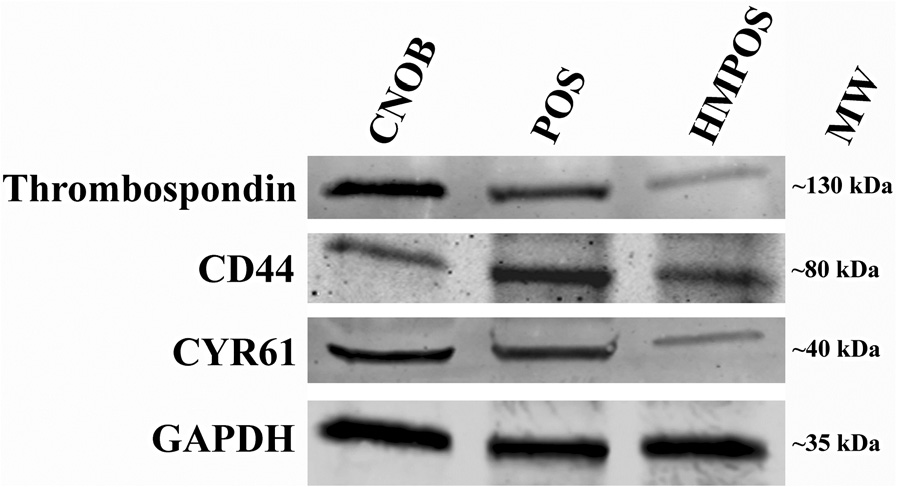
**Western blot of cell lines.** Western blot of whole cell lysate to detect the presence of CD44, Thrombospondin-1 and CYR61 in cultured normal canine osteoblasts (CnOb) and two validated canine osteosarcoma cell lines (POS and HMPOS).

Following validation and quantification of gene expression by qRT-PCR, five proteins were selected for further validation by Western blot: Thrombospondin-1, CYR61, CD44, Notch2 and Plexin B2. Due to the dearth of validated, canine-specific antibodies, these targets were selected based on the availability of antibodies that were likely to be cross-reactive with canine proteins. The antibodies against Notch2 and Plexin B2 were found to have poor target specificity towards the canine proteins in our samples and were excluded from the final figure. Western blots of whole cell lysates from all three cell lines was effective for Thrombospondin-1, CYR61 and CD44, and indicated that all three proteins were present in each cell line, albeit with differential abundance (Figure 3).

### Immunocytochemistry

**Figure 4:**
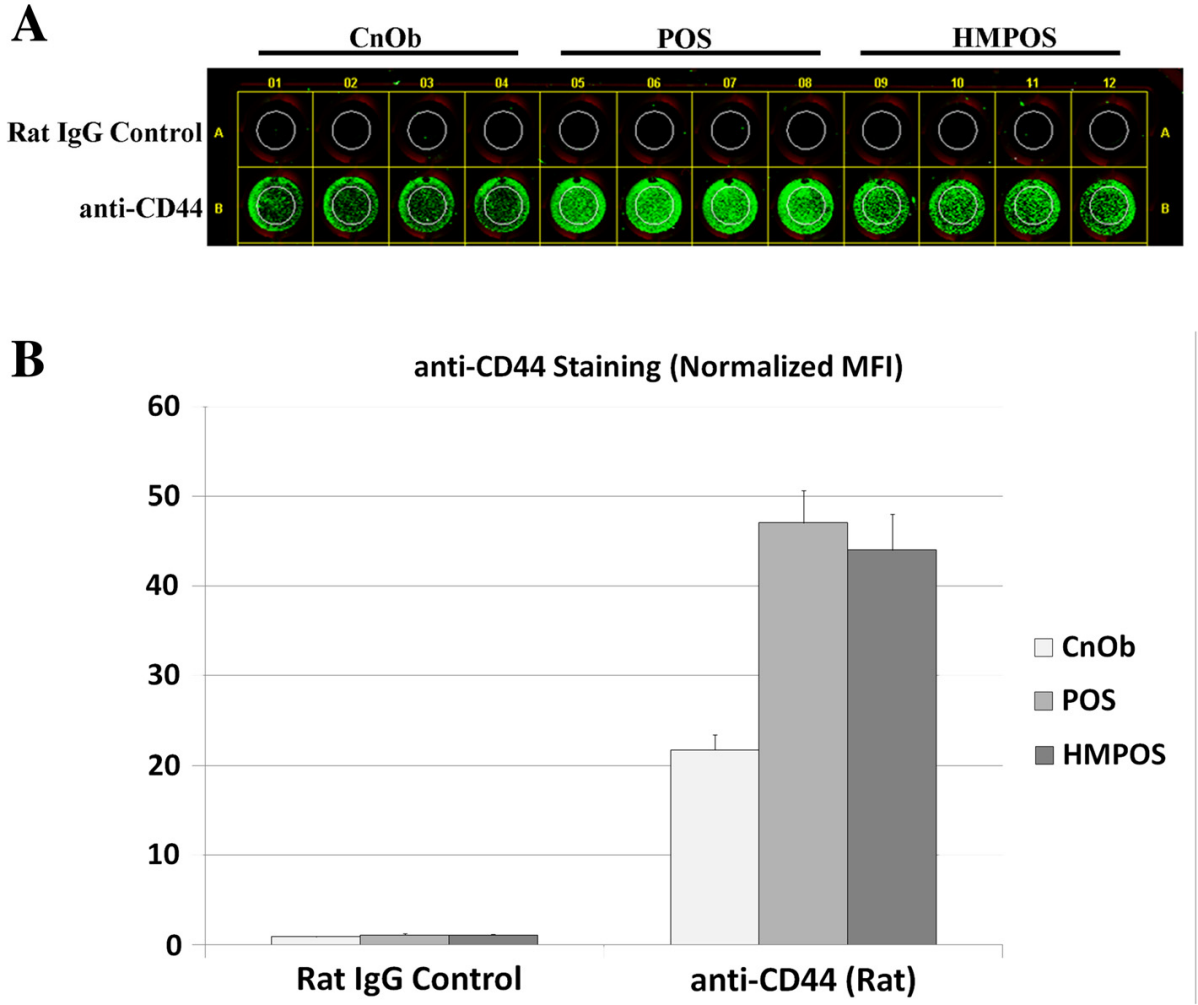
**anti-CD44 immunocytochemistry on live cell lines. A**. On-cell Western blot image showing binding of anti-CD44 antibody to the surface of live CnOb (normal canine osteoblast cell line), POS and HMPOS (canine osteosarcoma cell lines). **B**. Semi-quantitative analysis of CD44 density on cell surface indicating increased surface expression in both osteosarcoma cell lines (POS and HMPOS), relative to normal osteoblasts (CnOb). Quantification measures mean fluorescent intensity (MFI) of anti-CD44 staining, normalized to the approximate number of cells (quantified by nuclear stain).

To assess surface-localization of the selected proteins, an on-cell Western blot approach was used to visualize and quantify the proteins on the surface of live cells. Of the five experimental antibodies tested in this study, only the anti-CD44 antibody was validated for either immunocytochemistry or flow cytometry applications, and only with murine cells. Not surprisingly, only the anti-CD44 antibody was observed to bind, at detectable levels, to the cell surface of the cell lines used in this study. The fluorescent signal from anti-CD44 was quantified on a fluorescent scanner and normalized to the approximate number of cells, as measured by the signal intensity from the nuclear stain Hoecsht 33342. This semi-quantitative ICC analysis indicated that all three cell lines expressed CD44 on their surface, although it was much less abundant on the surface of CnOb cells compared to POS or HMPOS cells (Figure 4). The complete ICC results for all of the tested antibodies are summarized in Additional file 4.

## Discussion

The present study successfully employed a method of biotinylation/streptavidin enrichment with MS-based peptide sequencing to selectively label, purify, and identify surface exposed proteins from two validated canine OSA cell lines and a commercially available normal osteoblast line. A total of 133 putative surface-exposed proteins were identified when outcome from all cell lines was combined. Significant differences in SEP composition, and many similarities, were observed between the canine OSA cell lines (POS, HMPOS) and the normal canine osteoblast cell line (CnOb). Based on these observations, we conclude that MS can effectively detect SEP differences between cultured canine OSA and normal osteoblast cell lines.

When evaluating the list of 133 proteins identified via these methods, it is important to bear in mind that confident quantification of protein levels is not possible with this methodology because relative quantities can become skewed during the enrichment process, depending on the protein. Furthermore, this analysis invariably represents only a fraction of the total SEP; as a comprehensive profile would require the use of complementary sample processing methods, standards, and controls. Despite these limitations, the primary benefit of MS-based proteomic techniques, such as the one described in this report, is the ability to identify numerous previously unrecognized SEP differences between samples in a high-throughput fashion. This latitudinous analysis of the SEP can highlight novel targets for future diagnostic, prognostic and therapeutic methods [5,6,19].

MS-based proteomics is a relatively new tool in canine OSA research, and due to the limited information on this specific disease, we sought to validate a subset of our MS observations via secondary methods. These included qRT-PCR, Western blot, and immunocytochemistry. The results from these secondary tests were broadly confirmatory, but they occasionally contradictory, and highlighted some important limitations of the MS data sets. These results are summarized for a selected group of proteins in Table 2. For example, a negative result for an antigen in the MS data was not necessarily indicative that the target was absent, which was illustrated by our confirmation of CD44. Overall, the limited scope of this study means that the resulting data is inherently incomplete. However, these initial results also indicate that further improvements in sample processing methods, mass spectrometry technologies, and canine-specific antibodies will improve the feasibility and utility of this type of analysis.

**Table 2:** Summary of observations for selected surface proteins

**Protein name**	**Mass spec**	**qRT-PCR**	**Western blot**	**ICC**
Thrombospondin-1				
CnOb	D	High	High	N/A
POS	ND	Low	Mid	N/A
HMPOS	ND	Low	Low	N/A
CD44				
CnOb	ND	High	Low	Low
POS	D	Low	High	High
HMPOS	D	Low	Mid	High
CYR61				
CnOb	D	High	High	N/A
POS	D	Low	High	N/A
HMPOS	D	Low	Low	N/A
Plexin B2				
CnOb	ND	Low	N/A	N/A
POS	D	High	N/A	N/A
HMPOS	D	Mid	N/A	N/A
Notch-2				
CnOb	ND	Low	N/A	N/A
POS	D	Mid	N/A	N/A
HMPOS	D	High	N/A	N/A

A summary of observations for a subset of the surface-exposed proteins identified by mass spectrometry in cultured normal canine osteoblasts (CnOb) and two validated canine osteosarcoma cell lines (POS and HMPOS) and the results of their evaluation via secondary techniques (qRT-PCR, Western blot, and immunocytochemistry [ICC]).

*D* Detected.

*ND* Not detected.

High, Mid, Low = relative expression levels observed.

Our initial supportive experiment was qRT-PCR analysis of 11 of the 133 MS-identified surface-proteins. These 11 proteins were selected based on their known roles in cancer biology and apparent differences in abundance, as measured by MS spectral counts. Our analysis revealed that transcripts for all 11 genes were detected in each of the 3 cell lines, indicating that each of these genes were indeed expressed, and that each protein was potentially produced by each cell line. Protein quantification using spectral counting (label free approach) is considered to be semi-quantitative and the accuracy is based on the length of the protein, reproducibility of the sample preparation techniques, and LC separation [31]. In this study, because affinity enrichment was incorporated into the sample preparation, the relative abundance of the proteins determined by spectral counting can be misleading. Bearing this limitation in mind, the expression levels of several of the genes matched their apparent abundance at the cell surface, as measured by MS. Most notable among these was Thrombospondin-1, which was detected in unique abundance from CnOb samples, and whose transcript was also expressed at much higher levels in CnOb cells than in POS or HMPOS cells. At the same time, the apparent protein surface abundance did not closely match gene expression data for several of the targets. These differences may arise from differences in post-transcriptional regulation and/or protein trafficking to the cell surface, or they may be artifacts of the experimental method. While qRT-PCR demonstrates which genes are expressed and is an indicator of which proteins are likely to be more or less abundant, qRT-PCR is not a direct indicator of the abundance and localization of the corresponding proteins.

Western blot analysis demonstrated that the proteins Thrombospondin-1, CYR61 and CD44 were present at varying levels in all 3 cell lines. These results are compatible with the qRT-PCR results, indicating that these genes are both expressed and translated into protein in the cell lines studied. Again, the results were most consistent for Thrombospondin-1 protein, which was observed by Western Blot to be most abundant in the CnOb sample. Our MS analysis did not detect thombospondin-1 in the OSA cell lines, although the qRT-PCR and Western Blot results do indicate that this gene is transcribed and translated in the OSA cell lines, albeit at much lower levels than in CnOb. Additionally, CD44 was detected by qRT-PCR and Western blot in all three cell lines, but was not observed by MS in the CnOb cell line. The Western blot analysis represents all the proteins in the cells (whole cell lysate) whereas our MS-based SEP analysis is designed to specifically target the cell surface proteins. Therefore, the observed differences between Western blot results and MS findings may be due to changes in the protein localization with the cells, or they may represent limitations of our methods. Importantly, none of the proteins identified by MS were found to be absent in any cell line by Western blot (i.e. MS did not spuriously report presence of the three proteins, as verified by Western blot).

Immunocytochemistry represents the most direct method for confirming the surface localization of the proteins in question. However, the lack of validated antibodies for use with canine cells limits the population of proteins that could be confirmed using this technique. While all five of our antibodies were tested for potential reactivity, only one (αCD44) was found to bind to the surface of any of the cell lines, at a detectable level. This was result was not surprising because only the αCD44 was validated for flow cytometry applications. We observed positive CD44 staining for all of the cell lines, although the signal was much greater in the OSA lines compared to the CnOb cells. These results are largely consistent with our MS-based SEP analysis and are compatible with our Western blot observations. A previous study, using methods other than MS, has also demonstrated presence of CD44 on the cell surface of cultured canine OSA cells [32]. Furthermore, the CD44 immunocytochemistry findings suggest gene expression levels (assessed by qRT-PCR) and presence of CD44 in the whole cell lysate (assessed by Western blot) may not be indicative of concentration of the protein in question at the cellular surface—an observation that supports the selectivity of the biotin/streptavidin surface-protein enrichment technique employed in the present study.

Although the 133 proteins identified in this study represent a significant contribution to the study of canine OSA biology, they do not represent the entire SEP of these cells. A more comprehensive survey of the SEP composition could be achieved with the addition of complementary sample processing methods. For example, our method used amine-reactive biotinylation followed by Trypsin digestion of the recovered proteins to generate peptides for analysis. A more thorough SEP profile might incorporate an alternative biotinylation reagent (e.g. biotin hydrazide) [33] and/or protease (e.g. Glu-C) [34,35]. The addition of these methods would generate complementary data sets, ultimately increasing the depth, breadth, and accuracy of the analysis. Also, simply increasing the physical amount of sample analyzed for each cell line would be expected to yield more peptides for detection via MS, which would in turn increase the detection rate for proteins that exist in low quantities on the cell surface.

## Conclusions

In conclusion, the results of the present study identified numerous similarities and differences in the SEP between cultured canine OSA cell lines and cultured normal canine osteoblasts. The subset of these findings evaluated via secondary techniques including qRT-PCR, Western blot, and immunocytochemistry were found to be generally supportive of the mass spectrometry data, although the inconsistencies also demonstrate the limitations of this analysis (Table 2). These methods may be applied to other cell lines, or other biological materials, to highlight unique and previously unrecognized and unexpected differences between samples. While this study yielded data that may prove useful for OSA researchers and clinicians, further refinements of the described techniques are expected to yield greater accuracy and more comprehensive data sets.

## Methods

### Identification of surface-exposed proteins

The canine osteosarcoma cell lines POS and HMPOS [36] were cultured in RPMI 1640 medium supplemented with 10% fetal bovine serum and the normal canine osteoblast cell line, CnOb (Cell Applications, San Diego, CA), was cultured in canine osteoblast medium (Cell Applications, San Diego, CA). All cells were maintained in a humidified incubator with 5% CO_2_ at 37°C.

For all biotinylation experiments, cells were grown to ~80% confluency in T75 tissue culture flasks (Corning Inc, Corning, NY). Prior to biotinylation, cells were washed 5 times for 5 min with 10 mL Hanks Buffered Salt Solution (HBSS). The membrane-impermeable biotinylation reagent Sulfo-NHS-LC-Biotin (Pierce, Rockford, IL) was used to covalently label the surface-exposed proteins from live cultures of CnOb, POS and HMPOS cells: 5 mg of Sulfo-NHS-LC-Biotin (Pierce, Rockford, IL) was dissolved in 10 mL of HBSS and added to each flask. For negative controls (no biotinylation), 10 ml of plain HBSS was added to each flask. The biotinylation reaction was carried out in the tissue culture vessel, with gentle shaking, for 30 minutes at 4°C. Next, the biotinylation solution was removed and the reaction was quenched by the addition of HBSS supplemented with 10 mM glycine, which was incubated at room temperature for 20 minutes with gentle shaking. Cells were then washed twice with 10 mL of HBSS for 5 minutes at room temperature. Cell viability was verified on control samples with a live/dead stain (Invitrogen). For affinity purification experiments, the cells from each flask were harvested into 5 mL of Guanidinium Lysis Buffer (GLB) (140 mM NaCl, 10 mM KCl, 20 mM NaPhosphate, 10 mM EDTA, 2 mM EGTA, 0.1% Tween-20, 6 M Guanidinium HCl, pH 7.2). For Western Blot experiments, cells were harvested into 4 ml of RIPA buffer (Sigma, Saint Louis, MO) supplemented with protease inhibitor cocktail (Fermentas, Waltham, MA).

Insoluble debris in the whole cell lysate was pelleted by centrifugation for 10 minutes at 9,400 RCF at 4°C. DNA and other soluble biopolymers were removed by syringe filtration through a 0.2 μm filter. From each sample, 3 ml of lysate in GLB buffer was transferred to a clean tube and 100 μl of Streptavidin-coated C1 Dynabeads (Life Technologies, Grand Island, NY) was added to each tube. Samples were incubated at 23°C with agitation for 1 hr. Samples were washed twice for 10 minutes each with 1 ml of GLB and were then washed twice for 30 minutes each with WB-PBS (140 mM NaCl, 20 mM NaPhosphate, 0.05% Tween-20, pH 7.2). After washing, the supernatant was removed and 20 μl of RB solution (1% SDS in de-ionized H_2_0) was added and samples were incubated at 50°C for 10 minutes. After this initial incubation, 30 μl of Laemmli buffer with 5% BME (Bio-Rad) was added to each sample. Subsequently, samples were incubated at 50°C for 5 minutes and 70°C for 10 minutes. The Dynabeads were then pelleted by centrifugation and 40 μl of supernatant from each sample was used for SDS-PAGE. The capture and washing of the Dynabeads was performed with a magnetic stand (Life Technologies, Grand Island, NY).

To remove impurities, samples were run approximately 1 cm into a 12% SDS-PAGE gel (Bio-Rad, Hercules, CA). The proteins were then excised and digested in-gel using the ProteaseMAX/Trypsin gold system, following manufacturer protocols (Promega, Madison, WI). The resulting solution of eluted peptides was dehydrated to dryness by vacuum centrifugation at 23°C.

Proteomic analyses were performed in the Oregon State University MS facility and core. The LTQ-FT mass spectrometer was operated using data-dependent MS/MS acquisition with a MS precursor ion scan, performed in the ICR cell, from 350-2000 m/z with the resolving power set to 100,000 at m/z 400, and MS/MS scans performed by the linear ion trap on the five most abundant doubly or triply charged precursor ions detected in the MS scan. A binary solvent system consisting of solvent A, 2% aqueous acetonitrile with 0.1% formic acid, and solvent B, acetonitrile with 0.1% formic acid was used for the analyses. Tryptic peptides were loaded onto a peptide trapping column (Cap Trap, Michrom) and separated using a C18 column (Agilent Zorbax 300SB-C18, 250 x 0.3 mm, 5 μm). Peptides were trapped and washed with 3% solvent B for 3 min at a flow rate of 5 μL/min. Peptide separation was achieved using a linear gradient from 10% B to 30% B at a flow rate of 4 μL/min over 102 minutes. LC-MS/MS analysis was conducted on a Thermo LTQ-FT MS instrument coupled to a Waters nanoAcquity UPLC system.

Thermo RAW data files were processed with Proteome Discoverer v1.3.0. For database searching Mascot (v2.3) was used to search against the canine protein database downloaded from NCBI website (http://www.ncbi.nlm.nih.gov/). The following parameters were used for database searching: the digestion enzyme was set to Trypsin/P and two missed cleavage sites were allowed. The precursor ion mass tolerance was set to 10 ppm, while fragment ion tolerance of 0.8 Da was used. Dynamic modifications that were considered: carbamidomethyl (+57.02 Da) for cysteine and oxidation (+15.99 Da) for methionine, phosphorylation (+97.98 Da) of serine, threonine and tyrosine and biotinylation of lysine. Automatic target decoy search with 1% FRD was implemented into the Mascot search parameters. Scaffold_3.3.1 (Proteome Software, Portland, OR) was used for search data compilation and data evaluation with embedded X!Tandem database searching algorithm. For protein validation, peptide identification probability was set to 95% and protein identification probability was set to 99%, and proteins with two or more peptide matches were selected as true identifications [37].

### qRT-PCR

Cells were grown to ~80% confluency on 10 cm^2^ tissue culture plates, washed once with PBS and harvested into 1 mL of TRIzol reagent (Life Technologies, Grand Island, NY, Grand Island, NY). RNA was isolated by the manufacturer’s guidelines and the resulting RNA was quantified with the NanoDrop ND-1000 instrument. For cDNA synthesis 1000 ng of total RNA was reverse transcribed using Double Primed RNA to cDNA EcoDry™ Premix (Clontech, Mountain View, CA). All qRT-PCR assays were run in a total reaction volume of 10 μL comprised of 5 μL Power Sybr PCR Master Mix (Applied Biosystems, Carlsbad, CA), 500 nM of both forward and reverse primers and 1 μL of cDNA template. Real time PCR was performed in MicroAmp optical 96-well reaction plates using StepOnePlus Real-Time PCR Systems (Applied Biosystems, Carlsbad, CA). The cycle threshold (CT) for each gene of interest was normalized to GAPDH. The resulting delta-CT value was used to determine the relative quantification (RQ) of each gene using 2^(-delta CT). All RQ values were then divided by the RQ value of the highest expressing cell line and multiplied by 100 to determine relative differences in expression levels between the cell lines. All qRT-PCR primers were designed across exons in order to prevent background signal from any contaminating genomic DNA (Additional file 5: Table S5).

### Western blot

Insoluble debris in the cell lysate was pelleted by centrifugation for 10 minutes at 10,000 RPM at 4°C. The supernatant was then transferred to a new tube. Protein concentrations were assessed by BCA assay (Thermo Scientific, Rockford, IL). Approximately 5 μg of total protein was run in each lane. Proteins were denatured in Laemmli buffer with 5% BME (Bio-Rad, Hercules, CA) and separated by SDS-PAGE. Proteins were transferred to nitrocellulose films using the iBlot system (Life Technologies, Grand Island, NY). Primary antibodies targeting Thrombospondin-1 (sc-12312), CD44 (sc-18849), CYR61 (sc-8560), Notch 2 (sc-5545) and Plexin B2 (sc-34504) and GAPDH (sc-166574) (Santa Cruz Biotechnology, Santa Cruz, CA) were used at a dilution of 1:200 in PBS supplemented with 0.05% Tween-20 and 2% BSA. IRdye secondary antibodies (LI-COR, Lincoln, NE) were used at a dilution of 1:10,000 in PBS supplemented with 0.05% Tween-20 and 2% BSA. Western blots were visualized and recorded on the LI-COR scanning system (LI-COR, Lincoln, NE). To visualize biotinylated proteins, streptavidin IRDye680 (LI-COR, Lincoln, NE) was used for Western blot at a concentration of 1:10,000. To visualize total protein, samples were separated by SDS-PAGE and visualized by silver stain (Bio-Rad, Hercules, CA).

### Immunocytochemistry

Cells were cultured to approximately 80% confluency in a 96-well plate. Cells were incubated with primary antibodies (Rat IgG control (sc-2026), anti-CD44 (sc-18849), goat IgG control (sc-2028), anti-Thrombospondin-1 (sc-12312), CYR61 (sc-8560), anti-PlexinB2 (sc-34504), rabbit IgG control (sc-2027) and anti-Notch2 (sc- sc-5545) at a dilution of 1:200 in complete RPMI, at 4°C for 30 min (Santa Cruz Biotechnology). Cells were washed then incubated with secondary antibody, anti-rat-IgG-IRdye800 (926-32219), anti-goat-IgG-IRdye800 (926-32214) or anti-rabbit-IgG-IRdye800 (926-32211) a dilution of 1:1000 in complete RPMI, at 4°C for 30 min (LICOR). Cells were then washed extensively, the media replaced with 50 μl HBSS per well, and imaged on a LICOR scanner (LICOR). Hoescht 33342 (Sigma) was then added at a concentration of 2.5 μg/ml and incubated for 5 min at room temp. Cells were then washed and the fluorescent signal from the Hoeschst stain was read on a fluorescent plate reader. The fluorescent signal from the secondary antibodies was then normalized against the signal for Hoeschst, to account for differences in the number of cells (Additional file 4). Each condition had four technical replicates, which were used to establish the error bars (standard deviation).

## Availability of supporting data

The data sets supporting the results of this article are included within the article and its additional files.

## Competing interests

The author(s) declare that they have no competing interests.

## Authors’ contributions

All authors (MM, IHM, MJM, CPG, BS, SB, SIW) participated in conceptual study design, interpretation of the data, provided revisions to the manuscript, and read and approved the final manuscript. Additional individual author contributions are as follows: CPG and IHM performed the cell cultures; IHM and MJM performed the biotinylation and streptavidin purification; SIW performed the mass spectrometry analysis; MM, IHM, and MJM primarily authored the manuscript text. All authors read and approved the final manuscript.

## Supplementary Material

Additional file 1: Table S1 Mass spectrometry-identified cell surface-exposed proteins. Merged list of total cell surface-exposed protein biotinylation/streptavidin affinity purification mass spectrometry results for cultured normal canine osteoblasts (CnOb) and two validated canine osteosarcoma cell lines (POS and HMPOS). Click here for file

Additional file 2: Table S2 Biological replicates of mass spectrometry-identified cell surface-exposed proteins. Complete list, with each biological replicate, of identified cell surface-exposed protein biotinylation/streptavidin affinity purification mass spectrometry results for cultured normal canine osteoblasts (CnOb) and two validated canine osteosarcoma cell lines (POS and HMPOS). Click here for file

Additional file 3: Table S3 Biological replicates of quantitative real-time PCR data. Complete data, with each biological replicate, of quantitative real-time PCR results of cultured normal canine osteoblasts (CnOb) and two validated canine osteosarcoma cell lines (POS and HMPOS). Click here for file

Additional file 4 Primers used for quantitative real-time PCR of cultured normal canine osteoblasts (CnOb) and two validated canine osteosarcoma cell lines (POS and HMPOS). Click here for file

Additional file 5: Table S5 Immunocytochemistry data sets. Complete data, with all replicates and all tested antibodies, of immunocytochemistry (ICC) results for normal canine osteoblasts (CnOb) and two validated canine osteosarcoma cell lines (POS and HMPOS). Click here for file
