# Supplementary material for: Comparative analysis of the surface exposed proteome of two canine osteosarcoma cell lines and normal canine osteoblasts
Source: BMC Vet Res. 2013 Jun 13;9:116. doi: 10.1186/1746-6148-9-116 (PMC3684535; doi:10.1186/1746-6148-9-116)
Supplement: Additional file 1: Table S1 — Mass spectrometry-identified cell surface-exposed proteins. Merged list of total cell surface-exposed protein biotinylation/streptavidin affinity purification mass spectrometry results for cultured normal canine osteoblasts (CnOb) and two validated canine osteosarcoma cell lines (POS and HMPOS). [file 1746-6148-9-116-S1.pdf]

| # | Identified Proteins (122) | Accession Number | Molecular Weight | # of unique peptides | | |
|---|---|---|---|---|---|---|
| | | | | CNOB | HMPOS | POS |
| 23 | PREDICTED: thrombospondin-1 [Canis lupus familiaris] | gi\|345794639 | 130 kDa | 17 | 0 | 0 |
| 18 | PREDICTED: fibronectin [Canis lupus familiaris] | gi\|345797318 | 271 kDa | 16 | 4 | 6 |
| 39 | plasminogen activator inhibitor 1 precursor [Canis lupus familiaris] | gi\|308193314 | 45 kDa | 8 | 0 | 0 |
| 13 | glyceraldehyde-3-phosphate dehydrogenase [Canis lupus familiaris] | gi\|50978862 | 36 kDa | 5 | 9 | 5 |
| 15 | serpin peptidase inhibitor, clade H (heat shock protein 47), member 1, (collagen binding protein 1) [Canis lupus familiaris] | gi\|254674398 | 47 kDa | 5 | 5 | 10 |
| 21 | PREDICTED: prelamin-A/C isoform 4 [Canis lupus familiaris] | gi\|73960920 | 74 kDa | 4 | 10 | 4 |
| 2 | PREDICTED: tubulin alpha-1B chain [Canis lupus familiaris] | gi\|345792158 | 52 kDa | 3 | 13 | 14 |
| 10 | annexin 2 [Canis lupus familiaris] | gi\|37695552 | 39 kDa | 3 | 8 | 10 |
| 19 | PREDICTED: protein CYR61 [Canis lupus familiaris] | gi\|73960107 | 42 kDa | 3 | 13 | 1 |
| 36 | vitronectin [Canis lupus] | gi\|62421374 | 8 kDa | 2 | 2 | 3 |
| 59 | PREDICTED: integrin beta-1 [Canis lupus familiaris] | gi\|345793345 | 88 kDa | 2 | 0 | 4 |
| 77 | PREDICTED: actin, alpha cardiac muscle 1 isoform 3 [Canis lupus familiaris] | gi\|57108093 | 42 kDa | 2 | 0 | 1 |

| | | | | | | |
|---|---|---|---|---|---|---|
| 3 | PREDICTED: serine protease HTRA1 [Canis lupus familiaris] | gi\|345792549 | 41 kDa | 1 | 12 | 14 |
| 5 | elongation factor 1-alpha 1 [Canis lupus familiaris] | gi\|308199425 | 50 kDa | 1 | 12 | 7 |
| 12 | PREDICTED: tubulin beta-2C chain isoform 1 [Canis lupus familiaris] | gi\|73967439 | 50 kDa | 1 | 10 | 9 |
| 1 | PREDICTED: chondroitin sulfate proteoglycan 4 [Canis lupus familiaris] | gi\|345794811 | 245 kDa | 0 | 0 | 51 |
| 4 | PREDICTED: 60 kDa heat shock protein, mitochondrial [Canis lupus familiaris] | gi\|345797614 | 61 kDa | 0 | 13 | 20 |
| 6 | PREDICTED: pyruvate kinase isozymes M1/M2 isoform 1 [Canis lupus familiaris] | gi\|74000677 | 58 kDa | 0 | 18 | 15 |
| 7 | PREDICTED: ATP synthase subunit beta, mitochondrial isoform 1 [Canis lupus familiaris] | gi\|73968432 | 56 kDa | 0 | 11 | 16 |
| 8 | PREDICTED: LOW QUALITY PROTEIN: plexin-B2 [Canis lupus familiaris] | gi\|345777068 | 205 kDa | 0 | 5 | 20 |
| 9 | PREDICTED: heat shock protein HSP 90-alpha isoform 1 [Canis lupus familiaris] | gi\|359320163 | 78 kDa | 0 | 13 | 8 |
| 11 | PREDICTED: elongation factor 2 [Canis lupus familiaris] | gi\|359322142 | 95 kDa | 0 | 14 | 10 |
| 14 | PREDICTED: ATP synthase subunit alpha, mitochondrial isoform 2 [Canis lupus familiaris] | gi\|345802726 | 60 kDa | 0 | 8 | 14 |
| 16 | peroxiredoxin-1 [Canis lupus familiaris] | gi\|356461044 | 22 kDa | 0 | 6 | 7 |
| 17 | PREDICTED: heat shock cognate 71 kDa protein isoform 1 [Canis lupus familiaris] | gi\|57085907 | 71 kDa | 0 | 10 | 5 |
| 20 | PREDICTED: heat shock protein HSP 90-beta isoform 1 [Canis lupus familiaris] | gi\|359320981 | 83 kDa | 0 | 10 | 4 |
| 22 | PREDICTED: malate dehydrogenase, mitochondrial [Canis lupus familiaris] | gi\|73957776 | 35 kDa | 0 | 6 | 9 |

| | | | | | | |
|---|---|---|---|---|---|---|
| 24 | PREDICTED: ephrin type-A receptor 2 isoform 1 [Canis lupus familiaris] | gi\|73950854 | 108 kDa | 0 | 3 | 14 |
| 25 | PREDICTED: probable serine protease HTRA3 [Canis lupus familiaris] | gi\|345798114 | 53 kDa | 0 | 8 | 5 |
| 26 | PREDICTED: inactive tyrosine-protein kinase 7 isoform 1 [Canis lupus familiaris] | gi\|73972878 | 118 kDa | 0 | 1 | 16 |
| 27 | PREDICTED: protein disulfide-isomerase [Canis lupus familiaris] | gi\|73964749 | 57 kDa | 0 | 2 | 11 |
| 28 | eukaryotic initiation factor 4A-I [Canis lupus familiaris] | gi\|354623039 | 46 kDa | 0 | 4 | 6 |
| 29 | alkaline phosphatase [Canis lupus familiaris] | gi\|23267155 | 56 kDa | 0 | 5 | 6 |
| 30 | PREDICTED: ADP/ATP translocase 2 isoform 2 [Canis lupus familiaris] | gi\|74008194 | 33 kDa | 0 | 3 | 6 |
| 31 | PREDICTED: alpha-2-HS-glycoprotein isoform 1 [Canis lupus familiaris] | gi\|359323766 | 39 kDa | 0 | 3 | 3 |
| 32 | PREDICTED: dolichyl-diphosphooligosaccharide--protein glycosyltransferase subunit 1 isoform 3 [Canis lupus familiaris] | gi\|73984484 | 69 kDa | 0 | 1 | 9 |
| 33 | RecName: Full=Sodium/potassium-transporting ATPase subunit alpha-1; Short=Na/K ATPase alpha-1 subunit; AltName: Full=Sodium pump subunit alpha-1; Flags: Precursor | gi\|1703466 | 113 kDa | 0 | 0 | 11 |
| 34 | cyclophilin A [Canis lupus familiaris] | gi\|8699209 | 17 kDa | 0 | 4 | 8 |
| 35 | RecName: Full=Creatine kinase B-type; AltName: Full=B-CK; AltName: Full=Creatine kinase B chain | gi\|125292 | 43 kDa | 0 | 1 | 8 |
| 37 | PREDICTED: elongation factor 1-gamma isoform 1 [Canis lupus familiaris] | gi\|73983414 | 50 kDa | 0 | 2 | 6 |

| | | | | | | |
|---|---|---|---|---|---|---|
| 38 | PREDICTED: C-type mannose receptor 2, partial [Canis lupus familiaris] | gi\|345804924 | 162 kDa | 0 | 1 | 7 |
| 40 | PREDICTED: 78 kDa glucose-regulated protein isoform 5 [Canis lupus familiaris] | gi\|345806081 | 72 kDa | 0 | 3 | 7 |
| 41 | PREDICTED: fructose-bisphosphate aldolase A isoform 2 [Canis lupus familiaris] | gi\|73958481 | 40 kDa | 0 | 4 | 3 |
| 42 | ubiquitin-60S ribosomal protein L40 [Canis lupus familiaris] | gi\|224994158 | 15 kDa | 0 | 2 | 2 |
| 43 | PREDICTED: LOW QUALITY PROTEIN: alpha-enolase isoform 1 [Canis lupus familiaris] | gi\|345800677 | 49 kDa | 0 | 3 | 7 |
| 44 | PREDICTED: LOW QUALITY PROTEIN: protocadherin Fat 1 [Canis lupus familiaris] | gi\|345781801 | 507 kDa | 0 | 7 | 0 |
| 45 | unnamed protein product [Canis lupus familiaris] | gi\|207008756 | 29 kDa | 0 | 3 | 2 |
| 46 | PREDICTED: delta-sarcoglycan [Canis lupus familiaris] | gi\|345799473 | 32 kDa | 0 | 1 | 6 |
| 47 | PREDICTED: CD109 antigen isoform 3 [Canis lupus familiaris] | gi\|359321010 | 162 kDa | 0 | 0 | 6 |
| 48 | CD44 antigen precursor [Canis lupus familiaris] | gi\|308081450 | 40 kDa | 0 | 3 | 3 |
| 49 | PREDICTED: alpha-actinin-4 isoform 2 [Canis lupus familiaris] | gi\|73947718 | 105 kDa | 0 | 4 | 4 |
| 50 | PREDICTED: annexin A1 [Canis lupus familiaris] | gi\|73946797 | 39 kDa | 0 | 5 | 3 |
| 51 | PREDICTED: LOW QUALITY PROTEIN: heterogeneous nuclear ribonucleoprotein U isoform 1 [Canis lupus familiaris] | gi\|345802949 | 96 kDa | 0 | 3 | 2 |
| 52 | Chain A, Structure Of Full Length Grp94 With Amp-Pnp Bound | gi\|159794954 | 76 kDa | 0 | 2 | 4 |
| 53 | PREDICTED: T-complex protein 1 subunit beta isoform 1 [Canis lupus familiaris] | gi\|73968673 | 57 kDa | 0 | 4 | 2 |

| | | | | | | |
|---|---|---|---|---|---|---|
| 54 | PREDICTED: transitional endoplasmic reticulum ATPase isoform 3 [Canis lupus familiaris] | gi\|73971210 | 89 kDa | 0 | 2 | 2 |
| 55 | PREDICTED: poly(rC)-binding protein 2 isoform 1 [Canis lupus familiaris] | gi\|73996203 | 38 kDa | 0 | 2 | 4 |
| 56 | PREDICTED: sodium/potassium-transporting ATPase subunit alpha-3 [Canis lupus familiaris] | gi\|359318809 | 129 kDa | 0 | 1 | 3 |
| 57 | PREDICTED: glypican-4 [Canis lupus familiaris] | gi\|74008970 | 62 kDa | 0 | 0 | 3 |
| 58 | PREDICTED: 4F2 cell-surface antigen heavy chain [Canis lupus familiaris] | gi\|73983790 | 62 kDa | 0 | 0 | 3 |
| 60 | PREDICTED: cadherin-11 isoform 1 [Canis lupus familiaris] | gi\|73957344 | 88 kDa | 0 | 1 | 4 |
| 61 | PREDICTED: ATP-citrate synthase isoform 2 [Canis lupus familiaris] | gi\|73965857 | 121 kDa | 0 | 2 | 3 |
| 62 | PREDICTED: heterogeneous nuclear ribonucleoprotein H isoform 19 [Canis lupus familiaris] | gi\|73970381 | 49 kDa | 0 | 2 | 3 |
| 63 | L-lactate dehydrogenase B chain [Canis lupus familiaris] | gi\|356461040 | 37 kDa | 0 | 1 | 3 |
| 64 | PREDICTED: tubulin alpha-1A chain isoform 10 [Canis lupus familiaris] | gi\|345792156 | 50 kDa | 0 | 2 | 2 |
| 65 | galectin-1 [Canis lupus familiaris] | gi\|313766806 | 15 kDa | 0 | 0 | 4 |
| 66 | PREDICTED: neuropilin-1 isoform 2 [Canis lupus familiaris] | gi\|345793339 | 103 kDa | 0 | 0 | 6 |
| 67 | PREDICTED: voltage-dependent anion-selective channel protein 2 isoform 2 [Canis lupus familiaris] | gi\|73953093 | 32 kDa | 0 | 3 | 2 |
| 68 | PREDICTED: filamin-A isoform 9 [Canis lupus familiaris] | gi\|345807377 | 275 kDa | 0 | 1 | 3 |

| | | | | | | |
|---|---|---|---|---|---|---|
| 69 | PREDICTED: histone H4-like [Canis lupus familiaris] | gi\|345792378 | 11 kDa | 0 | 2 | 1 |
| 70 | PREDICTED: T-complex protein 1 subunit theta isoform 1 [Canis lupus familiaris] | gi\|74001080 | 60 kDa | 0 | 2 | 2 |
| 71 | PREDICTED: neurogenic locus notch homolog protein 2 isoform 1 [Canis lupus familiaris] | gi\|345782746 | 265 kDa | 0 | 1 | 5 |
| 72 | PREDICTED: fatty acid synthase [Canis lupus familiaris] | gi\|73964695 | 269 kDa | 0 | 0 | 4 |
| 73 | 40S ribosomal protein S4, X isoform [Canis lupus familiaris] | gi\|354725914 | 30 kDa | 0 | 3 | 1 |
| 74 | RecName: Full=Apolipoprotein A-I; Short=Apo-AI; Short=ApoA-I; AltName: Full=Apolipoprotein A1; Contains: RecName: Full=Truncated apolipoprotein A-I; Flags: Precursor | gi\|3915607 | 30 kDa | 0 | 2 | 1 |
| 75 | PREDICTED: cytoskeleton-associated protein 4 [Canis lupus familiaris] | gi\|73969959 | 65 kDa | 0 | 0 | 3 |
| 76 | PREDICTED: cysteine-rich secretory protein LCCL domain-containing 2 [Canis lupus familiaris] | gi\|345801034 | 56 kDa | 0 | 4 | 0 |
| 78 | PREDICTED: pleiotrophin [Canis lupus familiaris] | gi\|73978886 | 19 kDa | 0 | 0 | 3 |
| 79 | PREDICTED: vasorin [Canis lupus familiaris] | gi\|345802354 | 71 kDa | 0 | 1 | 4 |
| 80 | PREDICTED: EMILIN-1, partial [Canis lupus familiaris] | gi\|345781934 | 107 kDa | 0 | 3 | 1 |
| 81 | collagen alpha-3(VI) chain precursor [Canis lupus familiaris] | gi\|157151714 | 343 kDa | 0 | 0 | 5 |
| 82 | PREDICTED: collagen alpha-1(VI) chain [Canis lupus familiaris] | gi\|359323606 | 109 kDa | 0 | 0 | 5 |
| 83 | PREDICTED: serotransferrin isoform 1 [Canis lupus familiaris] | gi\|73990142 | 78 kDa | 0 | 3 | 0 |

| | | | | | | |
|---|---|---|---|---|---|---|
| 84 | heterogeneous nuclear ribonucleoprotein K [Canis lupus familiaris] | gi\|375065844 | 51 kDa | 0 | 1 | 2 |
| 85 | Chain i, Structure Of A Mammalian Ribosomal 60s Subunit Within An 80s Complex Obtained By Docking Homology Models Of The Rna And Proteins Into An 8.7 A Cryo-Em Map | gi\|187609307 | 18 kDa | 0 | 1 | 2 |
| 86 | plasminogen [Canis lupus familiaris] | gi\|18139619 | 41 kDa | 0 | 2 | 1 |
| 87 | PREDICTED: inter-alpha-trypsin inhibitor heavy chain H2 [Canis lupus familiaris] | gi\|73949158 | 107 kDa | 0 | 2 | 0 |
| 88 | PREDICTED: protein sidekick-2 [Canis lupus familiaris] | gi\|345804803 | 239 kDa | 0 | 0 | 3 |
| 89 | platelet glycoprotein IIIa [Canis lupus familiaris] | gi\|4206165 | 86 kDa | 0 | 0 | 3 |
| 90 | PREDICTED: hyaluronan and proteoglycan link protein 1 isoform 2 [Canis lupus familiaris] | gi\|73952134 | 40 kDa | 0 | 3 | 0 |
| 91 | alpha-fetoprotein [Canis lupus familiaris] | gi\|22218072 | 69 kDa | 0 | 2 | 0 |
| 92 | PREDICTED: T-complex protein 1 subunit alpha isoform 1 [Canis lupus familiaris] | gi\|57032236 | 60 kDa | 0 | 2 | 2 |
| 93 | PREDICTED: T-complex protein 1 subunit gamma isoform 1 [Canis lupus familiaris] | gi\|345802573 | 61 kDa | 0 | 1 | 2 |
| 94 | Chain b, Structure Of A Mammalian Ribosomal 60s Subunit Within An 80s Complex Obtained By Docking Homology Models Of The Rna And Proteins Into An 8.7 A Cryo-Em Map | gi\|187609300 | 46 kDa | 0 | 2 | 1 |
| 95 | BAT1 [Canis lupus familiaris] | gi\|39540669 | 49 kDa | 0 | 2 | 1 |
| 96 | Chain c, Structure Of A Mammalian Ribosomal 40s Subunit Within An 80s Complex Obtained By Docking Homology Models Of The Rna And Proteins Into An 8.7 | gi\|187609257 | 27 kDa | 0 | 3 | 0 |

| | | | | | | |
|---|---|---|---|---|---|---|
| | A Cryo-Em Map | | | | | |
| 97 | RecName: Full=Ras-related protein Rab-10 | gi\|131804 | 23 kDa | 0 | 0 | 2 |
| 98 | PREDICTED: tubulin beta chain [Canis lupus familiaris] | gi\|345778644 | 42 kDa | 0 | 2 | 0 |
| 99 | PREDICTED: neural cell adhesion molecule L1 isoform 1 [Canis lupus familiaris] | gi\|345807361 | 140 kDa | 0 | 0 | 2 |
| 100 | PREDICTED: bone morphogenetic protein 1-like [Canis lupus familiaris] | gi\|359322833 | 113 kDa | 0 | 0 | 4 |
| 101 | PREDICTED: carboxypeptidase Z [Canis lupus familiaris] | gi\|345798118 | 71 kDa | 0 | 0 | 3 |
| 102 | guanine nucleotide-binding regulatory protein alpha inhibitory subunit [Canis lupus familiaris] | gi\|45505263 | 41 kDa | 0 | 0 | 2 |
| 103 | PREDICTED: seprase isoform 1 [Canis lupus familiaris] | gi\|345797011 | 85 kDa | 0 | 0 | 2 |
| 104 | Na+/K+-exchanging ATPase (EC 3.6.3.9) alpha chain - dog (fragment) | gi\|104142 | 24 kDa | 0 | 0 | 2 |
| 105 | PREDICTED: neuroplastin [Canis lupus familiaris] | gi\|345794779 | 47 kDa | 0 | 0 | 2 |
| 106 | PREDICTED: fascin isoform 1 [Canis lupus familiaris] | gi\|345801349 | 55 kDa | 0 | 1 | 2 |
| 107 | PREDICTED: plasma membrane calcium-transporting ATPase 4 isoform 3 [Canis lupus familiaris] | gi\|74005825 | 129 kDa | 0 | 0 | 3 |
| 108 | PREDICTED: collagen alpha-2(VI) chain-like [Canis lupus familiaris] | gi\|359323618 | 87 kDa | 0 | 0 | 2 |
| 109 | pp90 precursor [Canis lupus familiaris] | gi\|1838958 | 68 kDa | 0 | 0 | 2 |

| | | | | | | |
|---|---|---|---|---|---|---|
| 110 | PREDICTED: serine hydroxymethyltransferase, mitochondrial isoform 2 [Canis lupus familiaris] | gi\|73968474 | 56 kDa | 0 | 0 | 2 |
| 111 | PREDICTED: protein disulfide-isomerase A4 isoform 3 [Canis lupus familiaris] | gi\|359321459 | 72 kDa | 0 | 0 | 2 |
| 112 | PREDICTED: d-3-phosphoglycerate dehydrogenase-like [Canis lupus familiaris] | gi\|359321669 | 57 kDa | 0 | 0 | 2 |
| 113 | PREDICTED: protein disulfide-isomerase A3 [Canis lupus familiaris] | gi\|345794865 | 57 kDa | 0 | 0 | 2 |
| 114 | PREDICTED: L-lactate dehydrogenase A chain isoform 1 [Canis lupus familiaris] | gi\|345787872 | 37 kDa | 0 | 0 | 2 |
| 115 | PREDICTED: ATP-dependent RNA helicase DDX3X isoform 3 [Canis lupus familiaris] | gi\|74006722 | 73 kDa | 0 | 3 | 0 |
| 116 | RecName: Full=Intercellular adhesion molecule 1; Short=ICAM-1; AltName: CD_antigen=CD54; Flags: Precursor | gi\|2506778 | 58 kDa | 0 | 2 | 0 |
| 117 | PREDICTED: clathrin heavy chain 1 isoform 1 [Canis lupus familiaris] | gi\|73966629 | 192 kDa | 0 | 0 | 2 |
| 118 | PREDICTED: heterogeneous nuclear ribonucleoprotein A1-like isoform 2 [Canis lupus familiaris] | gi\|73996139 | 34 kDa | 0 | 2 | 0 |
| 119 | putative secreted frizzled related protein 2 [Canis lupus familiaris] | gi\|30519782 | 33 kDa | 0 | 0 | 2 |
| 120 | PREDICTED: coagulation factor V [Canis lupus familiaris] | gi\|345803274 | 250 kDa | 0 | 2 | 0 |
| 121 | PREDICTED: importin subunit beta-1 isoform 1 [Canis lupus familiaris] | gi\|73966186 | 97 kDa | 0 | 0 | 2 |
| 122 | PREDICTED: heterogeneous nuclear ribonucleoprotein D0 isoform 2 [Canis lupus familiaris] | gi\|74001695 | 33 kDa | 0 | 2 | 0 |
