# Supplementary material for: Comparative analysis of the surface exposed proteome of two canine osteosarcoma cell lines and normal canine osteoblasts
Source: BMC Vet Res. 2013 Jun 13;9:116. doi: 10.1186/1746-6148-9-116 (PMC3684535; doi:10.1186/1746-6148-9-116)
Supplement: Additional file 2: Table S2 — Biological replicates of mass spectrometry-identified cell surface-exposed proteins. Complete list, with each biological replicate, of identified cell surface-exposed protein biotinylation/streptavidin affinity purification mass spectrometry results for cultured normal canine osteoblasts (CnOb) and two validated canine osteosarcoma cell lines (POS and HMPOS). [file 1746-6148-9-116-S2.pdf]

#	Identified Proteins (114)	Accession Number	Molecular Weight	# of unique peptides								
				CNOB-Rep1	CNOB-Rep2	CNOB-Rep3	HMPOS-Rep1	HMPOS-Rep2	HMPOS-Rep3	POS-Rep1	POS-Rep2	POS-Rep3
1	PREDICTED: chondroitin sulfate proteoglycan 4 [Canis lupus familiaris]	gi|345794811	245 kDa	0	0	0	0	0	0	8	42	45
2	PREDICTED: tubulin alpha-1B chain [Canis lupus familiaris]	gi|345792158	52 kDa	3	0	0	8	9	18	11	11	8
3	PREDICTED: serine protease HTRA1 [Canis lupus familiaris]	gi|345792549	41 kDa	0	0	0	5	9	8	8	13	14
4	PREDICTED: 60 kDa heat shock protein, mitochondrial [Canis lupus familiaris]	gi|345797614	61 kDa	0	0	0	11	3	9	14	15	10
5	elongation factor 1-alpha 1 [Canis lupus familiaris]	gi|308199425	50 kDa	1	0	0	4	6	13	5	4	9
6	PREDICTED: pyruvate kinase isozymes M1/M2 isoform 1 [Canis lupus familiaris]	gi|74000677	58 kDa	0	0	0	5	2	22	12	6	4
7	annexin 2 [Canis lupus familiaris]	gi|37695552	39 kDa	3	0	1	4	4	11	8	4	7
8	PREDICTED: LOW QUALITY PROTEIN: plexin-B2 [Canis lupus familiaris]	gi|345777068	205 kDa	0	0	0	3	1	0	3	18	17
9	PREDICTED: heat shock protein HSP 90-alpha isoform 1 [Canis lupus familiaris]	gi|359320163	78 kDa	0	0	0	5	6	19	3	3	4
10	PREDICTED: ATP synthase subunit beta, mitochondrial isoform 1 [Canis lupus familiaris]	gi|73968432	56 kDa	0	0	0	8	7	6	10	8	6
11	PREDICTED: glyceraldehyde-3-phosphate dehydrogenase-like [Canis lupus familiaris]	gi|345807932	36 kDa	5	1	0	4	6	11	5	4	3
12	PREDICTED: tubulin beta-2C chain isoform 1 [Canis lupus familiaris]	gi|73967439	50 kDa	1	0	0	8	6	10	6	8	7
13	PREDICTED: elongation factor 2 [Canis lupus familiaris]	gi|359322142	95 kDa	0	0	0	0	4	17	5	7	6
14	serpin peptidase inhibitor, clade H (heat shock protein 47), member 1, (collagen binding protein 1) [Canis lupus familiaris]	gi|254674398	47 kDa	3	0	1	5	3	5	8	7	5
15	PREDICTED: ATP synthase subunit alpha, mitochondrial isoform 2 [Canis lupus familiaris]	gi|345802726	60 kDa	0	0	0	3	2	7	6	8	7
16	peroxiredoxin-1 [Canis lupus familiaris]	gi|356461044	22 kDa	0	0	0	2	4	8	5	4	3
17	PREDICTED: ephrin type-A receptor 2 isoform 1 [Canis lupus familiaris]	gi|73950854	108 kDa	0	0	0	1	2	1	1	7	12
18	PREDICTED: protein CYR61 [Canis lupus familiaris]	gi|73960107	42 kDa	3	0	0	6	11	9	0	1	0
19	PREDICTED: heat shock protein HSP 90-beta isoform 1 [Canis lupus familiaris]	gi|359320981	83 kDa	0	0	0	2	2	11	2	2	4
20	PREDICTED: inactive tyrosine-protein kinase 7 isoform 1 [Canis lupus familiaris]	gi|73972878	118 kDa	0	0	0	1	0	0	0	10	12

21	PREDICTED: heat shock cognate 71 kDa protein isoform 1 [Canis lupus familiaris]	gi|57085907	71 kDa	0	0	0	4	3	13	3	1	1
22	PREDICTED: prelamin-A/C isoform 4 [Canis lupus familiaris]	gi|73960920	74 kDa	3	1	0	4	2	10	4	1	0
23	PREDICTED: malate dehydrogenase, mitochondrial [Canis lupus familiaris]	gi|73957776	35 kDa	0	0	0	2	5	4	7	7	4
24	PREDICTED: histone H2A type 1-E-like [Canis lupus familiaris]	gi|345796934	14 kDa	0	0	0	1	1	5	2	1	2
25	PREDICTED: fibronectin [Canis lupus familiaris]	gi|345797318	271 kDa	11	1	0	4	1	0	0	6	2
26	eukaryotic initiation factor 4A-I [Canis lupus familiaris]	gi|354623039	46 kDa	0	0	0	1	2	5	2	5	3
27	PREDICTED: probable serine protease HTRA3 [Canis lupus familiaris]	gi|345798114	53 kDa	0	0	0	1	5	4	1	5	2
28	alkaline phosphatase [Canis lupus familiaris]	gi|23267155	56 kDa	0	0	0	3	5	2	1	4	6
29	PREDICTED: dolichyl-diphosphooligosaccharide--protein glycosyltransferase subunit 1 isoform 3 [Canis lupus familiaris]	gi|73984484	69 kDa	0	0	0	0	0	1	2	6	9
30	PREDICTED: thrombospondin-1 [Canis lupus familiaris]	gi|345794639	130 kDa	13	7	1	0	0	0	0	0	0
31	RecName: Full=Sodium/potassium-transporting ATPase subunit alpha-1; Short=Na /K ATPase alpha-1 subunit; AltName: Full=Sodium pump subunit alpha-1; Flags: Precursor	gi|1703466	113 kDa	0	0	0	0	0	0	0	5	12
32	PREDICTED: ADP/ATP translocase 2 isoform 2 [Canis lupus familiaris]	gi|74008194	33 kDa	0	0	0	2	1	4	0	5	5
33	PREDICTED: protein disulfide-isomerase [Canis lupus familiaris]	gi|73964749	57 kDa	0	0	0	0	0	3	5	7	2
34	PREDICTED: elongation factor 1-gamma isoform 1 [Canis lupus familiaris]	gi|73983414	50 kDa	0	0	0	1	2	1	1	4	6
35	PREDICTED: alpha-2-HS-glycoprotein isoform 1 [Canis lupus familiaris]	gi|359323766	39 kDa	0	0	0	1	3	3	2	0	2
36	cyclophilin A [Canis lupus familiaris]	gi|8699209	17 kDa	0	0	0	0	2	5	6	0	0
37	PREDICTED: C-type mannose receptor 2, partial [Canis lupus familiaris]	gi|345804924	162 kDa	0	0	0	0	0	0	0	4	5
38	PREDICTED: 78 kDa glucose-regulated protein isoform 5 [Canis lupus familiaris]	gi|345806081	72 kDa	0	0	0	1	0	4	3	5	1
39	plasminogen activator inhibitor 1 precursor [Canis lupus familiaris]	gi|308193314	45 kDa	8	3	1	0	0	0	0	0	0
40	RecName: Full=Creatine kinase B-type; AltName: Full=B-CK; AltName: Full=Creatine kinase B chain	gi|125292	43 kDa	0	0	0	0	0	1	6	3	3
41	vitronectin [Canis lupus]	gi|62421374	8 kDa	1	0	1	1	2	1	2	2	1
42	PREDICTED: fructose-bisphosphate aldolase A isoform 2 [Canis lupus familiaris]	gi|73958481	40 kDa	0	0	0	1	1	6	2	0	0
43	PREDICTED: CD109 antigen isoform 3 [Canis lupus familiaris]	gi|359321010	162 kDa	0	0	0	0	0	0	0	4	5
44	unnamed protein product [Canis lupus familiaris]	gi|207008756	29 kDa	0	0	0	1	1	3	1	0	1
45	RecName: Full=CD44 antigen	gi|2499844	38 kDa	0	0	0	2	0	0	0	3	2

46	PREDICTED: annexin A1 [Canis lupus familiaris]	gi|73946797	39 kDa	0	0	0	1	1	7	1	0	2
47	PREDICTED: LOW QUALITY PROTEIN: alpha-enolase isoform 1 [Canis lupus familiaris]	gi|345800677	49 kDa	0	0	0	0	0	3	5	1	0
48	PREDICTED: LOW QUALITY PROTEIN: protocadherin Fat 1 [Canis lupus familiaris]	gi|345781801	507 kDa	0	0	0	2	5	0	0	1	2
49	ubiquitin-60S ribosomal protein L40 [Canis lupus familiaris]	gi|224994158	15 kDa	0	0	0	2	0	3	1	2	2
50	PREDICTED: alpha-actinin-4 isoform 2 [Canis lupus familiaris]	gi|73947718	105 kDa	0	0	0	1	0	4	2	1	1
51	PREDICTED: delta-sarcoglycan [Canis lupus familiaris]	gi|345799473	32 kDa	0	0	0	0	0	0	1	6	4
52	PREDICTED: T-complex protein 1 subunit beta isoform 1 [Canis lupus familiaris]	gi|73968673	57 kDa	0	0	0	1	1	2	1	2	0
53	PREDICTED: sodium/potassium-transporting ATPase subunit alpha-3 [Canis lupus familiaris]	gi|359318809	129 kDa	0	0	0	0	1	0	1	3	3
54	PREDICTED: LOW QUALITY PROTEIN: heterogeneous nuclear ribonucleoprotein U isoform 1 [Canis lupus familiaris]	gi|345802949	96 kDa	0	0	0	1	0	3	1	1	0
55	Chain A, Structure Of Full Length Grp94 With Amp-Pnp Bound	gi|159794954	76 kDa	0	0	0	2	0	1	1	2	2
56	PREDICTED: ATP-citrate synthase isoform 2 [Canis lupus familiaris]	gi|73965857	121 kDa	0	0	0	1	0	2	1	1	2
57	PREDICTED: glypican-4 [Canis lupus familiaris]	gi|74008970	62 kDa	0	0	0	0	0	0	1	3	3
58	L-lactate dehydrogenase B chain [Canis lupus familiaris]	gi|356461040	37 kDa	0	0	0	0	0	2	1	2	1
59	PREDICTED: poly(rC)-binding protein 2 isoform 1 [Canis lupus familiaris]	gi|73996203	38 kDa	0	0	0	1	1	2	2	3	0
60	PREDICTED: cadherin-11 isoform 1 [Canis lupus familiaris]	gi|73957344	88 kDa	0	0	0	0	1	0	1	3	3
61	PREDICTED: integrin beta-1 [Canis lupus familiaris]	gi|345793345	88 kDa	1	0	0	0	0	0	0	3	3
62	PREDICTED: 4F2 cell-surface antigen heavy chain [Canis lupus familiaris]	gi|73983790	62 kDa	0	0	0	0	0	0	0	1	3
63	PREDICTED: neuropilin-1 isoform 2 [Canis lupus familiaris]	gi|345793339	103 kDa	0	0	0	0	0	0	0	5	5
64	PREDICTED: fatty acid synthase [Canis lupus familiaris]	gi|73964695	269 kDa	0	0	0	0	0	0	0	4	1
65	RecName: Full=Apolipoprotein A-I; Short=Apo-AI; Short=ApoA-I; AltName: Full=Apolipoprotein A1; Contains: RecName: Full=Truncated apolipoprotein A-I; Flags: Precursor	gi|3915607	30 kDa	0	0	0	1	2	2	1	1	1
66	PREDICTED: voltage-dependent anion-selective channel protein 2 isoform 2 [Canis lupus familiaris]	gi|73953093	32 kDa	0	0	0	1	2	2	0	2	0
67	PREDICTED: heterogeneous nuclear ribonucleoprotein H isoform 19 [Canis lupus familiaris]	gi|73970381	49 kDa	0	0	0	0	0	2	0	2	1
68	PREDICTED: tubulin alpha-1A chain isoform 10 [Canis lupus familiaris]	gi|345792156	50 kDa	0	0	0	1	1	2	1	1	2

69	PREDICTED: T-complex protein 1 subunit theta isoform 1 [Canis lupus familiaris]	gi|74001080	60 kDa	0	0	0	2	0	2	1	2	1
70	40S ribosomal protein S4, X isoform [Canis lupus familiaris]	gi|354725914	30 kDa	0	0	0	1	0	3	1	1	0
71	PREDICTED: histone H4-like [Canis lupus familiaris]	gi|345792378	11 kDa	0	0	0	0	1	3	0	0	0
72	PREDICTED: neurogenic locus notch homolog protein 2 isoform 1 [Canis lupus familiaris]	gi|345782746	265 kDa	0	0	0	1	0	0	0	5	1
73	PREDICTED: cysteine-rich secretory protein LCCL domain-containing 2 [Canis lupus familiaris]	gi|345801034	56 kDa	0	0	0	3	2	0	0	0	0
74	PREDICTED: cytoskeleton-associated protein 4 [Canis lupus familiaris]	gi|73969959	65 kDa	0	0	0	0	0	0	1	3	1
75	PREDICTED: pleiotrophin [Canis lupus familiaris]	gi|73978886	19 kDa	0	0	0	0	0	0	2	2	1
76	PREDICTED: vasorin [Canis lupus familiaris]	gi|345802354	71 kDa	0	0	0	1	0	0	0	3	1
77	galectin-1 [Canis lupus familiaris]	gi|313766806	15 kDa	0	0	0	0	0	1	2	1	0
78	PREDICTED: collagen alpha-1(VI) chain [Canis lupus familiaris]	gi|359323606	109 kDa	0	0	0	0	0	0	0	5	2
79	Chain i, Structure Of A Mammalian Ribosomal 60s Subunit Within An 80s Complex Obtained By Docking Homology Models Of The Rna And Proteins Into An 8.7 A Cryo-Em Map	gi|187609307	18 kDa	0	0	0	0	1	1	2	0	0
80	PREDICTED: filamin-A isoform 9 [Canis lupus familiaris]	gi|345807377	275 kDa	0	0	0	0	0	1	0	0	2
81	collagen alpha-3(VI) chain precursor [Canis lupus familiaris]	gi|157151714	343 kDa	0	0	0	0	0	0	0	4	2
82	PREDICTED: serotransferrin isoform 1 [Canis lupus familiaris]	gi|73990142	78 kDa	0	0	0	3	2	0	0	0	0
83	platelet glycoprotein IIIa [Canis lupus familiaris]	gi|4206165	86 kDa	0	0	0	0	0	0	0	3	1
84	Chain b, Structure Of A Mammalian Ribosomal 60s Subunit Within An 80s Complex Obtained By Docking Homology Models Of The Rna And Proteins Into An 8.7 A Cryo-Em Map	gi|187609300	46 kDa	0	0	0	0	0	2	1	0	0
85	PREDICTED: neuroplastin [Canis lupus familiaris]	gi|345794779	47 kDa	0	0	0	0	0	0	0	2	1
86	PREDICTED: neutral amino acid transporter B(0) isoform 1 [Canis lupus familiaris]	gi|73947234	57 kDa	0	0	0	1	0	0	0	1	2
87	RecName: Full=Ras-related protein Rab-10	gi|131804	23 kDa	0	0	0	0	0	0	1	1	2
88	PREDICTED: heterogeneous nuclear ribonucleoprotein L-like [Canis lupus familiaris]	gi|359318672	64 kDa	0	0	0	0	0	3	0	0	0
89	Chain c, Structure Of A Mammalian Ribosomal 40s Subunit Within An 80s Complex Obtained By Docking Homology Models Of The Rna And Proteins Into An 8.7 A Cryo-Em Map	gi|187609257	27 kDa	0	0	0	0	1	3	0	0	0
90	PREDICTED: protein sidekick-2 [Canis lupus familiaris]	gi|345804803	239 kDa	0	0	0	0	0	0	0	3	2
91	alpha-fetoprotein [Canis lupus familiaris]	gi|22218072	69 kDa	0	0	0	1	2	0	0	0	0

92	PREDICTED: hyaluronan and proteoglycan link protein 1 isoform 2 [Canis lupus familiaris]	gi|73952134	40 kDa	0	0	0	1	2	0	0	0	0
93	PREDICTED: bone morphogenetic protein 1-like [Canis lupus familiaris]	gi|359322833	113 kDa	0	0	0	0	0	0	0	2	1
94	PREDICTED: fascin isoform 1 [Canis lupus familiaris]	gi|345801349	55 kDa	0	0	0	0	0	1	2	0	0
95	BAT1 [Canis lupus familiaris]	gi|39540669	49 kDa	0	0	0	0	0	2	1	0	0
96	PREDICTED: inter-alpha-trypsin inhibitor heavy chain H2 [Canis lupus familiaris]	gi|73949158	107 kDa	0	0	0	0	2	1	0	0	0
97	pp90 precursor [Canis lupus familiaris]	gi|1838958	68 kDa	0	0	0	0	0	0	0	2	2
98	PREDICTED: seprase isoform 1 [Canis lupus familiaris]	gi|345797011	85 kDa	0	0	0	0	0	0	0	2	2
99	PREDICTED: collagen alpha-2(VI) chain-like [Canis lupus familiaris]	gi|359323618	87 kDa	0	0	0	0	0	0	0	1	2
100	PREDICTED: plasma membrane calcium-transporting ATPase 4 isoform 3 [Canis lupus familiaris]	gi|74005825	129 kDa	0	0	0	0	0	0	0	0	3
101	PREDICTED: carboxypeptidase Z [Canis lupus familiaris]	gi|345798118	71 kDa	0	0	0	0	0	0	0	3	0
102	PREDICTED: integrin alpha-V isoform 2 [Canis lupus familiaris]	gi|74004771	109 kDa	0	0	0	0	0	0	0	2	1
103	PREDICTED: ATP-dependent RNA helicase DDX3X isoform 3 [Canis lupus familiaris]	gi|74006722	73 kDa	0	0	0	0	0	3	0	0	0
104	PREDICTED: polypyrimidine tract-binding protein 1 isoform 1 [Canis lupus familiaris]	gi|73987389	60 kDa	0	0	0	0	0	2	1	0	0
105	PREDICTED: importin-5 isoform 1 [Canis lupus familiaris]	gi|359322489	121 kDa	0	0	0	0	0	0	0	0	2
106	PREDICTED: heterogeneous nuclear ribonucleoprotein A1-like isoform 2 [Canis lupus familiaris]	gi|73996139	34 kDa	0	0	0	0	0	2	0	0	0
107	putative secreted frizzled related protein 2 [Canis lupus familiaris]	gi|30519782	33 kDa	0	0	0	0	0	0	0	2	0
108	PREDICTED: matrix-remodeling-associated protein 8 [Canis lupus familiaris]	gi|345800581	50 kDa	0	0	0	0	0	0	0	2	0
109	PREDICTED: coagulation factor V [Canis lupus familiaris]	gi|345803274	250 kDa	0	0	0	0	2	0	0	0	0
110	PREDICTED: trifunctional enzyme subunit alpha, mitochondrial-like [Canis lupus familiaris]	gi|359321587	83 kDa	0	0	0	0	0	0	0	2	0
111	PREDICTED: profilin-1-like [Canis lupus familiaris]	gi|73955372	18 kDa	0	0	0	0	0	2	0	0	0
112	PREDICTED: inosine-5'-monophosphate dehydrogenase 2 isoform 5 [Canis lupus familiaris]	gi|73985592	53 kDa	0	0	0	0	0	2	0	0	0
113	PREDICTED: dolichyl-diphosphooligosaccharide--protein glycosyltransferase subunit 2 isoform 3 [Canis lupus familiaris]	gi|73991908	69 kDa	0	0	0	0	0	0	0	0	2
114	PREDICTED: heterogeneous nuclear ribonucleoprotein D0 isoform 2 [Canis lupus familiaris]	gi|74001695	33 kDa	0	0	0	0	0	2	0	0	0
