# Supplementary material for: Comparative analysis of the surface exposed proteome of two canine osteosarcoma cell lines and normal canine osteoblasts
Source: BMC Vet Res. 2013 Jun 13;9:116. doi: 10.1186/1746-6148-9-116 (PMC3684535; doi:10.1186/1746-6148-9-116)

Sample Name	Target Name	cT	dCT	RQ	RQ average	Percent of highest expressor
CNOB #1	CD44	23.13586	2.016206	0.247207	0.192388	128.4942
CNOB #2	CD44	21.0001	2.465452	0.181061		94.11242
CNOB #3	CD44	21.46163	2.747622	0.148896		77.39363
HMPOS #1	CD44	23.00928	5.204315	0.027123		14.09831
HMPOS #2	CD44	23.79644	5.395113	0.023763		12.35183
HMPOS #3	CD44	23.33386	5.318661	0.025057		13.02404
POS #1	CD44	23.17706	4.960126	0.032126		16.69842
POS #2	CD44	22.44697	4.295809	0.050913		26.46395
POS #3	CD44	22.72163	4.401646	0.047312		24.59205
CNOB #1	CRISP	32.41168	11.29203	0.000399		1.293601
CNOB #2	CRISP	29.50583	10.97118	0.000498		1.615792
CNOB #3	CRISP	29.34538	10.63137	0.00063		2.044929
HMPOS #1	CRISP	22.83478	5.029814	0.030611		99.29234
HMPOS #2	CRISP	23.35318	4.951854	0.03231		104.8055
HMPOS #3	CRISP	23.1626	5.147403	0.028215		91.5204
POS #1	CRISP	23.3139	5.096968	0.029219	0.030829	94.77644
POS #2	CRISP	23.25063	5.099467	0.029168		94.61238
POS #3	CRISP	23.1941	4.874121	0.034099		110.6074
CNOB #1	CSPG4	28.53679	7.417137	0.005851	0.006764	86.49992
CNOB #2	CSPG4	25.87884	7.344185	0.006154		90.9864
CNOB #3	CSPG4	25.62913	6.915125	0.008286		122.5001
HMPOS #1	CSPG4	30.82357	13.01861	0.000121		1.781579
HMPOS #2	CSPG4	29.51386	11.11254	0.000452		6.677113
HMPOS #3	CSPG4	29.63965	11.62445	0.000317		4.682608
POS #1	CSPG4	26.56912	8.352187	0.00306		45.24156
POS #2	CSPG4	25.687	7.535838	0.005389		79.66785
POS #3	CSPG4	25.01224	6.692256	0.00967		142.964
CNOB #1	Ephrin	25.07185	3.952203	0.064605		8.465948
CNOB #2	Ephrin	21.60244	3.067783	0.119263		15.62832
CNOB #3	Ephrin	21.70168	2.987675	0.126072		16.52066
HMPOS #1	Ephrin	18.32865	0.52369	0.69559		91.15084
HMPOS #2	Ephrin	19.4233	1.021976	0.492442		64.53003
HMPOS #3	Ephrin	19.18371	1.168519	0.444878		58.29723
POS #1	Ephrin	18.88903	0.672096	0.627594	0.76312	82.24055
POS #2	Ephrin	18.73118	0.580022	0.668954		87.66035
POS #3	Ephrin	18.33039	0.010409	0.992811		130.0989
CNOB #1	Fibronectin	16.88421	-4.23544	18.83627	12.14606	155.0813
CNOB #2	Fibronectin	15.49002	-3.04464	8.251387		67.93468
CNOB #3	Fibronectin	15.48896	-3.22505	9.350533		76.98408
HMPOS #1	Fibronectin	19.08379	1.278823	0.412132		3.39313
HMPOS #2	Fibronectin	19.17725	0.77593	0.584012		4.808241
HMPOS #3	Fibronectin	19.06694	1.051746	0.482384		3.971526
POS #1	Fibronectin	21.19233	2.975397	0.12715		1.046841
POS #2	Fibronectin	20.4989	2.347744	0.196453		1.617422
POS #3	Fibronectin	20.5229	2.202916	0.217198		1.788219
CNOB #1	GAPDH	21.11965				
CNOB #2	GAPDH	18.53465				
CNOB #3	GAPDH	18.71401				
HMPOS #1	GAPDH	17.80496				
HMPOS #2	GAPDH	18.40132				
HMPOS #3	GAPDH	18.01519				
POS #1	GAPDH	18.21693				
POS #2	GAPDH	18.15116				
POS #3	GAPDH	18.31998				
CNOB #1	Neuropilin	30.41339	9.293736	0.001593		19.15525
CNOB #2	Neuropilin	28.19921	9.664563	0.001232		14.8135
CNOB #3	Neuropilin	28.49608	9.782075	0.001136		13.65473
HMPOS #1	Neuropilin	24.60546	6.800492	0.008971		107.8522
HMPOS #2	Neuropilin	25.20691	6.805588	0.00894		107.4719
HMPOS #3	Neuropilin	25.16452	7.149323	0.007044		84.68774
POS #1	Neuropilin	26.06575	7.84882	0.004338		52.14959

POS #2	Neuropilin	25.70851	7.557349	0.005309		63.82521
POS #3	Neuropilin	25.30078	6.980798	0.007917		95.18128
CNOB #1	Notch	22.89121	1.771556	0.292893		94.01296
CNOB #2	Notch	20.12842	1.593764	0.331306		106.3429
CNOB #3	Notch	20.40164	1.687634	0.310436		99.64388
HMPOS #1	Notch	19.82154	2.016578	0.247144		79.32843
HMPOS #2	Notch	20.46591	2.064587	0.239055		76.73203
HMPOS #3	Notch	20.19525	2.180057	0.220667		70.82993
POS #1	Notch	21.30671	3.089778	0.117458		37.70191
POS #2	Notch	21.31149	3.160326	0.111853		35.90263
POS #3	Notch	20.80105	2.481064	0.179112		57.49162

Sample	Target	cT	dCT	RQ	RQ averag	percent of highest expressor
CNOB #1	CD109	23.42008	5.447416	0.022917		47.25226
CNOB #2	CD109	21.47563	6.122423	0.014354		29.59547
CNOB #3	CD109	21.69626	6.068279	0.014903		30.72728
HMPOS #1	CD109	21.05168	5.888552	0.01688		34.80387
HMPOS #2	CD109	21.49851	6.099602	0.014583		30.06735
HMPOS #3	CD109	20.91004	5.490641	0.022241		45.85754
POS #1	CD109	21.00885	4.417347	0.0468	0.048517	96.49489
POS #2	CD109	20.85791	4.137163	0.056832		117.1785
POS #3	CD109	20.43959	4.5762	0.04192		86.43401
CNOB #1	CYR61	20.72338	2.750723	0.148576	0.130259	114.0623
CNOB #2	CYR61	18.43419	3.080976	0.118177		90.72478
CNOB #3	CYR61	18.63929	3.011307	0.124024		95.21352
HMPOS #1	CYR61	20.22349	5.060356	0.02997		23.0077
HMPOS #2	CYR61	19.98961	4.590702	0.041501		31.86055
HMPOS #3	CYR61	20.8294	5.409998	0.02352		18.05596
POS #1	CYR61	21.63343	5.041927	0.030355		23.30349
POS #2	CYR61	20.51461	3.793861	0.0721		55.35109
POS #3	CYR61	21.62322	5.759823	0.018455		14.16814
CNOB #1	Thrombospondi	18.92333	0.950666	0.517393	0.615512	84.05903
CNOB #2	Thrombospondi	15.82233	0.469124	0.722403		117.3662
CNOB #3	Thrombospondi	16.34883	0.720854	0.606738		98.57456
HMPOS #1	Thrombospondi	21.28823	6.125095	0.014327		2.327695
HMPOS #2	Thrombospondi	22.98775	7.588842	0.005194		0.843906
HMPOS #3	Thrombospondi	22.00548	6.586081	0.010409		1.691047
POS #1	Thrombospondi	20.87632	4.284813	0.051303		8.335013
POS #2	Thrombospondi	20.20524	3.484489	0.089344		14.51535
POS #3	Thrombospondi	20.17234	4.308945	0.050452		8.196753
CNOB #1	GAPDH	17.97266				
CNOB #2	GAPDH	15.35321				
CNOB #3	GAPDH	15.62798				
HMPOS #1	GAPDH	15.16313				
HMPOS #2	GAPDH	15.39891				
HMPOS #3	GAPDH	15.4194				
POS #1	GAPDH	16.5915				
POS #2	GAPDH	16.72075				
POS #3	GAPDH	15.86339				
CNOB #1	PLEXIN	28.36173	10.38907	0.000746		1.462208
CNOB #2	PLEXIN	22.22058	6.867369	0.008565		16.79368
CNOB #3	PLEXIN	22.52238	6.894402	0.008406		16.48193
HMPOS #1	PLEXIN	20.39164	5.228504	0.026672		52.29898
HMPOS #2	PLEXIN	20.77953	5.380619	0.024003		47.06544
HMPOS #3	PLEXIN	20.47197	5.05257	0.030132		59.08192
POS #1	PLEXIN	20.63165	4.040146	0.060785	0.05106	119.1859
POS #2	PLEXIN	20.99886	4.278107	0.051542		101.0628
POS #3	PLEXIN	20.47677	4.613379	0.040854		80.1059

averages

	CnOb	POS	HMPOS
CD44	100.0001	22.58481	13.15806
CRISP	1.651441	99.99873	98.53941
Ephrin 2a	13.53831	99.99994	71.32603
Fibronectir	100	1.484161	4.057632
Neuropilin	15.87449	70.38536	100.0039
Notch	15.87449	70.38536	100.0039
CD109	35.85834	100.0358	36.90959
CYR61	100.0002	30.94091	24.30807
Thrombosp	99.99994	10.34904	1.620883
Plexin 2b	11.57927	100.1182	52.81545
CSPG4	99.99547	89.29113	4.380433

standard deviations

	CnOb	POS	HMPOS
CD44	26.0541	5.18297	0.880921
CRISP	0.37693	9.187713	6.674472
Ephrin 2a	4.415396	26.20695	17.44934
Fibronectir	47.9159	0.388239	1.974926
Neuropilin	2.899694	22.25328	13.26559
Notch	6.172655	11.97883	4.355085
CD109	9.883638	15.67514	8.102964
CYR61	12.38323	21.62768	6.993559
Thrombosp	16.69928	3.608796	0.744378
Plexin 2b	8.763022	19.5571	6.024866
CSPG4	19.61824	49.56686	2.461716

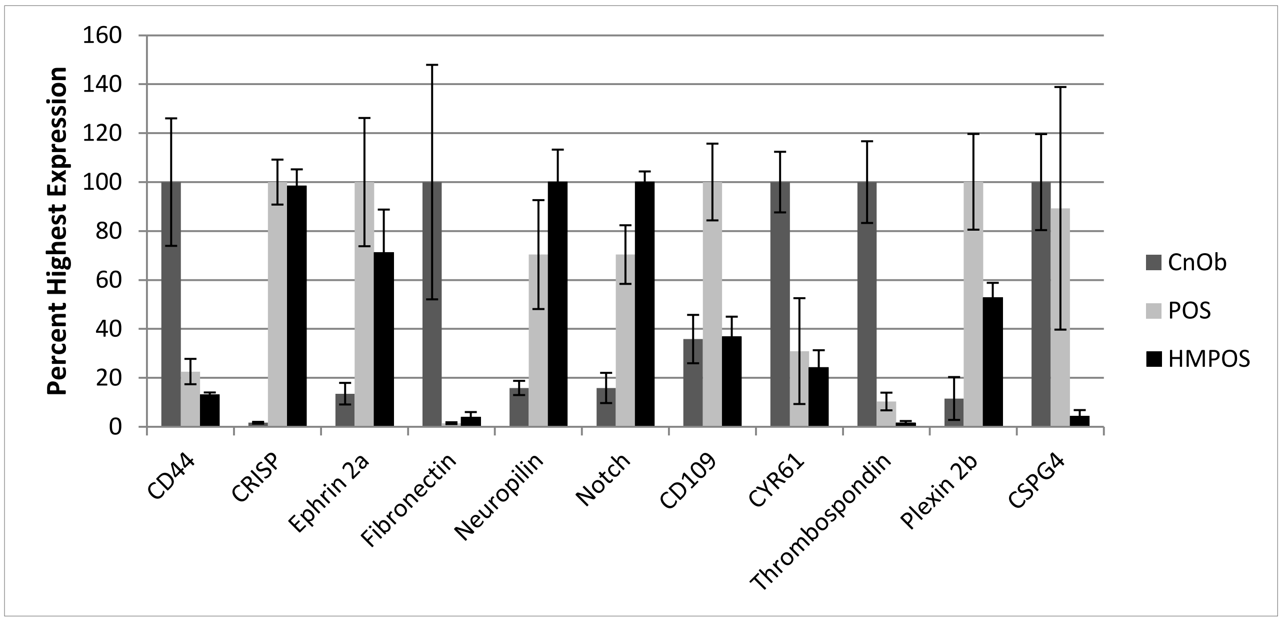

Supplement: Additional file 3: Table S3 — Biological replicates of quantitative real-time PCR data. Complete data, with each biological replicate, of quantitative real-time PCR results of cultured normal canine osteoblasts (CnOb) and two validated canine osteosarcoma cell lines (POS and HMPOS). [file 1746-6148-9-116-S3.pdf]
