# Supplementary material for: Comparative analysis of the surface exposed proteome of two canine osteosarcoma cell lines and normal canine osteoblasts
Source: BMC Vet Res. 2013 Jun 13;9:116. doi: 10.1186/1746-6148-9-116 (PMC3684535; doi:10.1186/1746-6148-9-116)

Surface Marker MFI (IRDye800-conjugated Secondary Ab)

		CnOb	CnOb	CnOb	CnOb	POS	POS	POS	POS	HMPOS	HMPOS	HMPOS	HMPOS
		1	2	3	4	5	6	7	8	9	10	11	12
Rat IgG Control	A	0.74	0.82	0.89	0.75	0.88	1.07	1.17	1.22	0.89	0.94	0.88	0.92
anti-CD44 (Rat)	B	20.12	17.01	18.58	19.92	42.74	50.2	48.43	44.91	34.28	37.01	39.92	33.09
Goat IgG Control	C	1.05	1.05	0.78	0.97	0.84	1.6	0.91	0.86	0.98	0.85	6.5	0.93
anti-Thrombospondir	D	0.82	2.4	1.18	0.93	0.86	1.29	1.04	4.78	6.08	3.17	4.33	0.97
anti-CYR61 (goat)	E	0.96	0.77	0.67	0.75	0.81	1.28	0.87	1.04	0.91	1.23	0.92	0.78
anti-PlexinB2 (goat)	F	0.89	1.05	0.73	0.96	1.49	1.01	0.93	1.6	1.14	0.9	0.74	0.69
Rabbit IgG control	G	1.09	0.98	0.67	0.76	2.31	1.6	1.77	0.73	0.69	0.72	0.71	0.66
anti-NOTCH2 (rabbit)	H	0.94	0.84	0.99	1.75	0.75	0.79	1.7	0.79	1.48	0.67	0.64	0.7

Nucleus Marker MFI (Abs/Emission = 365/460)

		CnOb	CnOb	CnOb	CnOb	POS	POS	POS	POS	HMPOS	HMPOS	HMPOS	HMPOS
		1	2	3	4	5	6	7	8	9	10	11	12
Rat IgG Control	A	860.449	850.381	957.79	907.183	927.116	1139.23	963.507	1019.56	802.839	821.393	817.417	939.896
anti-CD44 (Rat)	B	902.882	829.961	915.448	834.639	1002.37	976.869	1018.53	962.911	811.959	802.55	824.984	841.35
Goat IgG Control	C	809.096	792.512	812.346	1173.89	986.864	1212.17	1085.77	995.772	818.698	818.464	820.693	760.999
anti-Thrombospondir	D	772.093	819.726	836.943	1099.17	917.614	1013.95	1084.44	955.283	792.96	802.744	795.897	783.072
anti-CYR61 (goat)	E	722.716	819.744	755.119	830.366	899.015	1036.04	1007.84	966.902	764.702	862.765	792.097	756.522
anti-PlexinB2 (goat)	F	781.195	816.825	799.14	809.978	865.342	1129.9	982.44	995.208	753.262	787.739	810.356	769.588
Rabbit IgG control	G	716.049	792.636	749.218	861.094	1086.79	1128.12	1055.02	972.169	743.799	772.988	790.524	753.601
anti-NOTCH2 (rabbit)	H	775.033	787.978	786.796	915	871.586	959.336	957.879	894.806	809.455	782.225	775.961	781.097

Ratio Surface Marker/Nucleus Marker

		CnOb	CnOb	CnOb	CnOb	POS	POS	POS	POS	HMPOS	HMPOS	HMPOS	HMPOS
		1	2	3	4	5	6	7	8	9	10	11	12
Rat IgG Control	A	0.00086	0.000964	0.000929	0.000827	0.000949	0.000939	0.001214	0.001197	0.001109	0.001144	0.001077	0.000979
anti-CD44 (Rat)	B	0.022284	0.020495	0.020296	0.023867	0.042639	0.051389	0.047549	0.04664	0.042219	0.046116	0.048389	0.03933
Goat IgG Control	C	0.001298	0.001325	0.00096	0.000826	0.000851	0.00132	0.000838	0.000864	0.001197	0.001039	0.00792	0.001222
anti-Thrombospondir	D	0.001062	0.002928	0.00141	0.000846	0.000937	0.001272	0.000959	0.005004	0.007667	0.003949	0.00544	0.001239
anti-CYR61 (goat)	E	0.001328	0.000939	0.000887	0.000903	0.000901	0.001235	0.000863	0.001076	0.00119	0.001426	0.001161	0.001031
anti-PlexinB2 (goat)	F	0.001139	0.001285	0.000913	0.001185	0.001722	0.000894	0.000947	0.001608	0.001513	0.001143	0.000913	0.000897
Rabbit IgG control	G	0.001522	0.001236	0.000894	0.000883	0.002126	0.001418	0.001678	0.000751	0.000928	0.000931	0.000898	0.000876
anti-NOTCH2 (rabbit)	H	0.001213	0.001066	0.001258	0.001913	0.000861	0.000823	0.001775	0.000883	0.001828	0.000857	0.000825	0.000896

Ratio Surface Marker/Nucleus Marker (adjusted X 1,000)

		CnOb	CnOb	CnOb	CnOb	POS	POS	POS	POS	HMPOS	HMPOS	HMPOS	HMPOS
		1	2	3	4	5	6	7	8	9	10	11	12
Rat IgG Control	A	0.860016	0.964274	0.929222	0.826735	0.94918	0.939231	1.214314	1.196595	1.108566	1.144397	1.076562	0.978832
anti-CD44 (Rat)	B	22.2842	20.49494	20.29607	23.86661	42.63895	51.38867	47.54892	46.63982	42.21888	46.11551	48.38882	39.32965
Goat IgG Control	C	1.297745	1.324901	0.960182	0.826313	0.851181	1.319947	0.838115	0.863652	1.197023	1.038531	7.920136	1.222078
anti-Thrombospondir	D	1.062048	2.927808	1.409893	0.846093	0.937213	1.272252	0.95902	5.003753	7.667474	3.948955	5.440402	1.238711
anti-CYR61 (goat)	E	1.328323	0.939318	0.887277	0.903216	0.900986	1.235474	0.863232	1.0756	1.190006	1.425649	1.161474	1.031034
anti-PlexinB2 (goat)	F	1.13928	1.285465	0.913482	1.185217	1.721863	0.893884	0.946623	1.607704	1.513418	1.14251	0.913179	0.896584
Rabbit IgG control	G	1.522242	1.236381	0.894266	0.882598	2.125526	1.418289	1.677693	0.750898	0.92767	0.93145	0.898138	0.875795
anti-NOTCH2 (rabbit)	H	1.212852	1.06602	1.258268	1.912568	0.8605	0.823486	1.774754	0.882873	1.828391	0.856531	0.824784	0.896176

Average (MFI)

	CnOb	POS	HMPOS
Rat IgG Cor	0.895062	1.07483	1.077089
anti-CD44 (	21.73545	47.05409	44.01321
Goat IgG C(	1.102285	0.968224	2.844442
anti-Throm	1.56146	2.04306	4.573886
anti-CYR61	1.014533	1.018823	1.202041
anti-PlexinI	1.130861	1.292518	1.116423
Rabbit IgG	1.133872	1.493102	0.908263
anti-NOTCH	1.362427	1.085403	1.10147

Standard Deviation

	CnOb	POS	HMPOS
Rat IgG Cor	0.06286	0.15106	0.071124
anti-CD44 (	1.678652	3.59159	4.029928
Goat IgG C(	0.247734	0.234714	3.384772
anti-Throm	0.940043	1.979721	2.697843
anti-CYR61	0.210323	0.171518	0.164352
anti-PlexinI	0.157249	0.432909	0.287473
Rabbit IgG	0.306534	0.5746	0.026274
anti-NOTCH	0.375826	0.460219	0.485493

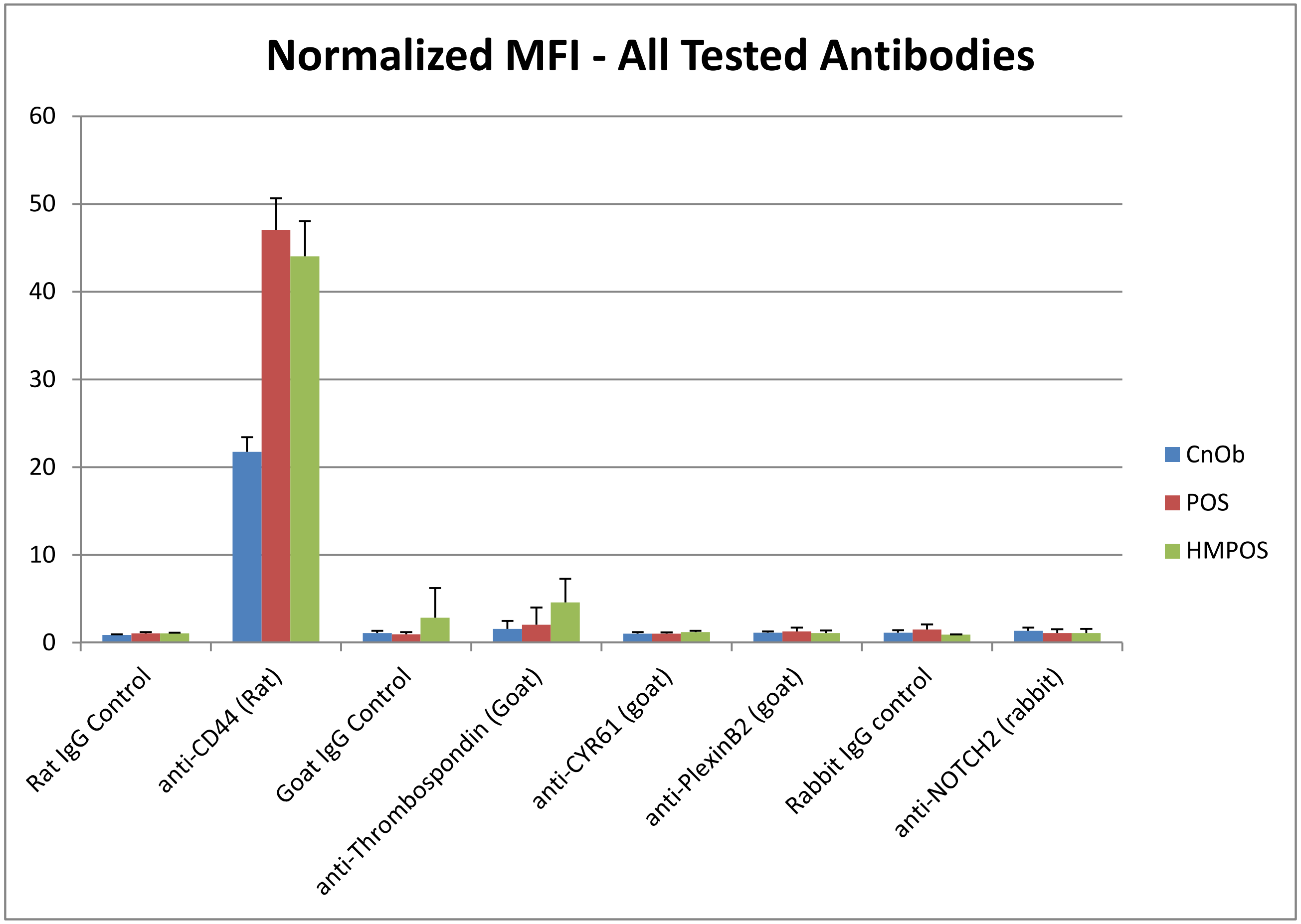

Supplement: Additional file 4 — Primers used for quantitative real-time PCR of cultured normal canine osteoblasts (CnOb) and two validated canine osteosarcoma cell lines (POS and HMPOS). [file 1746-6148-9-116-S4.pdf]
