# Supplementary material for: Comparative analysis of the surface exposed proteome of two canine osteosarcoma cell lines and normal canine osteoblasts
Source: BMC Vet Res. 2013 Jun 13;9:116. doi: 10.1186/1746-6148-9-116 (PMC3684535; doi:10.1186/1746-6148-9-116)
Supplement: Additional file 5: Table S5 — Immunocytochemistry data sets. Complete data, with all replicates and all tested antibodies, of immunocytochemistry (ICC) results for normal canine osteoblasts (CnOb) and two validated canine osteosarcoma cell lines (POS and HMPOS). [file 1746-6148-9-116-S5.pdf]

Supplemental Table 1: qRT-PCR Primers

Control Genes:

GAPDHcanFor: TATGTTGTGGAGTCCACTGGG
GAPDHcanRev: TCTTCTGGGTGGCAGTGATGG
Size: 280 bp

Experimental Genes:

CD44canFor: ATCCTCATATCCAACACCTCC
CD44canRev: GCTCCTTTCACTGGAGGATCC
Size: 246

CD109canFor: **ATGATCATGGAAAACTCAACC**
CD109canRev: AACTCCTCACCGCTTGTCTCC
Size: 159 bp

CSPG4canFor: AGGTCCACGACGCCCTGGAGC
CSPG4canRev: GCCGGGAACCGCGTCACCTGG
Product Size: 262 bp

CRISPLD2canFor: TCCTGGACAGCAAAGGTGGCC
CRISPLD2canRev: TGGCTCGTCTTTGCAGTGTGC
Size: 245

EPHA2canFor: CCTCGACAAGCTCATCCGCGC
EPHA2canRev: TCGTCGTTGGTCATCTGCACC
Size: 219

FN1canFor: CTCGGTGCTATTTGCTCCTGC
FN1canRev: CGGGAATCTTCTCTGTCAGCC
Size: 215 bp

NOTCH2canFor: TGTTGGGGAAAGCAACATGCC
NOTCH2canRev: GGTACAGGTACTTCCATTCGC
Size: 230 bp

NRP1canFor: GACCAGTTAGTCTGGATGGCC
NRP1canRev: CCTGGAGATGTTCTCATCACC
Size: 291 bp

PLXNB2canFor: ACGCCTGCTACACCGGCTCCC
PLXNB2canRev: TACTCCGCGGAGGTGCCGTCC
Size: 309

CYR61canFor: CACACCAAGGGGCTGGAATGC
CYR61canRev: GGGGAGCGAGAGCTCTTGGGG
Size: 210 bp

THBS1canFor: TCCAGAGCATCTTCACCAGGG
THBS1canRev: TGTGGCCGATGTAGTTAGTGC
Product Size: 237
